# Epicutaneous Sensitization and Food Allergy: Preventive Strategies Targeting Skin Barrier Repair—Facts and Challenges

**DOI:** 10.3390/nu15051070

**Published:** 2023-02-21

**Authors:** Anna Dębińska, Barbara Sozańska

**Affiliations:** Department and Clinic of Paediatrics, Allergology and Cardiology Wroclaw Medical University, ul. Chałubińskiego 2a, 50-368 Wrocław, Poland

**Keywords:** food allergy, epicutaneous sensitization, moisturizers, skin barrier, filaggrin

## Abstract

Food allergy represents a growing public health and socio-economic problem with an increasing prevalence over the last two decades. Despite its substantial impact on the quality of life, current treatment options for food allergy are limited to strict allergen avoidance and emergency management, creating an urgent need for effective preventive strategies. Advances in the understanding of the food allergy pathogenesis allow to develop more precise approaches targeting specific pathophysiological pathways. Recently, the skin has become an important target for food allergy prevention strategies, as it has been hypothesized that allergen exposure through the impaired skin barrier might induce an immune response resulting in subsequent development of food allergy. This review aims to discuss current evidence supporting this complex interplay between the skin barrier dysfunction and food allergy by highlighting the crucial role of epicutaneous sensitization in the causality pathway leading to food allergen sensitization and progression to clinical food allergy. We also summarize recently studied prophylactic and therapeutic interventions targeting the skin barrier repair as an emerging food allergy prevention strategy and discuss current evidence controversies and future challenges. Further studies are needed before these promising strategies can be routinely implemented as prevention advice for the general population.

## 1. Introduction

Food allergy is defined as an adverse, hypersensitivity reaction developing as a result of a specific immune response which is repeatable after re-exposure to a given food [1]. Despite the difficulties in obtaining exact prevalence data, there is agreement that food allergy has increased in the last 10–15 years and we are now riding a food allergy epidemic with an estimated frequency of 3% to 10% in high-income countries [2,3]. Moreover, recent findings indicate that severity of childhood food allergy is higher than previously assumed, as an estimated 42% of children with food allergy had a history of at least one severe, life-threatening allergic reaction and about 40% declared multiple food allergies [4,5]. Food allergies represent a considerable public health and socio-economic issue, but above all can have a profound negative impact on quality of life of affected individuals and their families [2,6,7,8]. The substantial psychological burden is further increased by the absence of a cure and limited treatment options, largely based on strict allergens avoidance and management of allergic reactions, the risk of which is significant due to ubiquitous presence of allergenic foods in our setting. Considering the aforementioned facts, the better understanding of the food allergy pathogenesis in order to develop effective preventive strategies that can stop this epidemic seems to be essential.

The pathogenesis of food allergy is a multifactorial process, influenced by genetic, epigenetic and environmental risk factors [9,10,11]. While several hypothesis have been proposed, it is now recognized that it is the complex interplay between genetic inheritance, diet, skin exposure and gut microbiome that is crucial to the development of food allergy [12,13,14]. Numerous studies suggest that skin barrier plays an essential role in the pathogenesis of food allergy through its ability to induce epicutaneous sensitization which corresponds to one half of the widely accepted dual allergen exposure hypothesis [12,15]. Although, the exact pathways leading from epicutaneuos food sensitization to clinical food allergy need to be elucidated, the skin is emerging as an important target for food allergy prevention strategies. Therefore, interventions targeting the skin barrier repair to prevent atopic dermatitis and food allergy have gained a lot of interest in recent years.

The purpose of this review is to discuss the mechanisms underlying epicutaneous sensitization to food allergens and risk factors influencing this process as well as current experimental and clinical evidence suggesting that impairment of the epidermal barrier initiates an immune response leading to subsequent development of food allergy. We also review current knowledge of topical intervention to improve skin barrier function as a strategy for prevention of food allergy, including moisturizer therapy and new topical therapy targeting upregulation of skin barrier-related molecules, such as filaggrin (FLG). We also discussed the challenges associated with such strategies.

## 2. The Dual Allergen Exposure Hypothesis

The dual allergen exposure hypothesis is one of the most extensively studied in the context of the development of food allergy, postulating that the timing and route of exposure to foods allergens lead to sensitization or tolerance [15,16]. In line with this hypothesis, early life, low-dose allergen exposure through the skin, especially impaired or inflamed as occurs in eczema, induces allergic sensitization and leads to subsequent food allergy, whereas early high-dose oral exposure to food allergens through the gastrointestinal tract promotes immune tolerance and prevent food allergy [17,18].

The conception of early oral introduction of allergenic foods into the diet to induce immune tolerance is derived from the observation that allergen avoidance, instead of reducing the incidence of food allergies, contributed to its increase in children, as well as from interventional studies on dietary elimination that have failed to decrease the rate of food allergy [17,19]. This is also supported by data from murine model showing that food allergen exposure is necessary to promote both immunologic and clinical tolerance to ingested food proteins [12,17,20]. There is growing evidence from clinical trials and meta-analyses that inducing oral tolerance by early introduction of allergenic foods into the infant’s diet is effective in preventing food allergy [19,21,22,23,24,25]. However, this strategy has been recognized and appears robust primarily for peanuts and eggs in a high-risk populations of children with severe atopic dermatitis or other food allergies [26,27,28,29,30,31]. Recent studies have suggested that it might be also the case with early introduction of cow’s milk protein [32,33]. Although the protective effect of early food administration is likely to be plausible for further allergenic foods, the sufficient evidence supporting its effectiveness for foods other than peanuts and eggs or at the general population level is currently lacking. In addition, this preventive strategy faces many limitations [34,35]. Evidence indicates that the induction of oral tolerance is allergen specific and requires the introduction of allergenic food over the well-defined time window that is possibly different for different foods [34,36]. This means the need of introducing multiple foods into the infant’s diet on complicated schedule and within the narrow “window of opportunity” which together constitutes a challenge for most families and hinders compliance. Furthermore, some food allergy may develop very early in life before the infant reaches the developmental age to introduce solid foods [27,37]. Additionally, the induction of oral tolerance may be stopped by the simultaneous exposure of the disturbed skin to environmental allergens [38,39]. Thus, there is still a need for alternative, easily achievable ways to prevent food allergy, which draws attention to the second part of the dual allergen hypothesis.

Evidence supporting the concept of the impaired skin barrier as a rout of allergic sensitization to food comes from numerous studies showing that children with atopic dermatitis have a significantly higher risk of food sensitization compared to healthy control [40]. Population-based studies reported the food sensitization rate in children with atopic dermatitis ranging between 55% to 66%, while the prevalence of food allergy confirmed by challenge reaches up to 81% [40]. Interestingly, in infants with atopic dermatitis sensitization to food allergens has been observed very early, even before oral consumption of these foods [27,41]. Furthermore, early onset, severity and chronicity of atopic dermatitis have been identified as strong predictors of food allergy [40,42,43]. In the Australia HealthNuts cohort the 6-fold increase in the risk of developing food allergy to egg white, peanut or sesame by 12 months of age was observed in infants with atopic dermatitis, particularly in those manifested the early-onset and severe atopic dermatitis [44]. Additionally, it was found that atopic dermatitis predates the development of food sensitization and allergy in most cases, suggesting the causal association and indicating that skin barrier dysfunction play a crucial role in the progression to food allergy [40,45,46]. The data from the MACS study indicated that atopic dermatitis in the first 6 months preceded and predicted the development of new-onset food sensitization within the first 2 years of life in previously unsensitized infants [47]. The primary defect that drives atopic dermatitis is the epidermal barrier impairment, consistently observed in both lesional and nonlesional skin, which is supposed to allow penetration of allergen and induce the subsequent systemic Th2 immune responses as a result of the phenomenon referred as epicutaneous sensitization [15,39,48,49].

## 3. The Concept of Epicutaneous Food Allergen Sensitization

The epicutaneous sensitization is a process strongly dependent on impaired skin barrier function, which promotes increased penetration of allergens stimulating Th2-mediated immune response and IgE production and consequently predisposes to the development of respiratory and/or food allergies [12,13,50]. Skin barrier is the primary interface between the organisms and the external environment. The healthy skin contributes to effective protection against harmful environmental factors, preserving the organism integrity and adapting its physiology to changing environments [51,52]. Except to provide the mechanical outside-inside barrier, the skin play important homeostatic, immune and sensory functions including regulation of body temperature, defense against microbial infection, activation of the innate antioxidant system and immune response, production of cytokines, hormones and neurotransmitters [53,54,55]. The most important defensive function of the skin is to construct the permeability barrier that allows to maintain the homeostasis by preventing excessive transcutaneous loss of water, electrolytes and serum proteins and also protects against the ingress of environmental irritants, allergens and pathogens [50,56].

**Figure 1:**
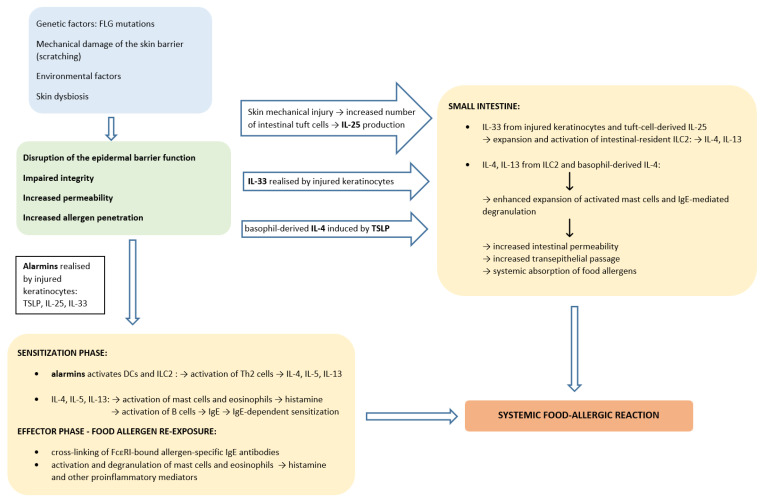
Mechanisms of epicutaneous sensitization and possible immune pathways associated with the progression to food allergy.

A disturbed skin barrier not only facilitates the penetration of allergens, but also releases the epithelium-derived cytokines (TSLP, IL-25, IL-33) which mediate Th2 response in an antigen independent manner by activating dendritic cells (DCs) and innate lymphoid cells type 2 (ILC2) [57,58]. In epicutaneous sensitization, resident DCs capture allergens passing through the disrupted epidermal barrier, present processed allergens to naïve CD4+ T cells in draining lymph nodes and prime differentiation of naïve CD4+ T cells into allergen-specific Th2 cells secreting proallergic cytokines (IL-4, IL-5, IL-9, IL-13) [59,60]. ILC2 also play an important role in food allergic sensitization by producing IL-4, IL-13 and IL-5 and modulating activity of DCs, Th2 cells, eosinophils and in particular mast cells [57,61,62]. IL-4 and IL-13 promote B-cell isotype switching to specific IgE cells that further differentiate into plasma cells producing large amount of allergen-specific IgE antibodies that bind to high-affinity FcεRI receptors on the surface of mast cells and basophils leading to a state of allergic sensitization. Subsequently, facilitated allergen presentation further drives the production of a memory pool of allergen-specific B cells and Th2 cells [63,64]. Following re-exposure to previously sensitizing allergens results in cross-linking of FcεRI-bound sIgE antibodies and activates mast cells and basophils degranulation, leading to the release of inflammatory mediators triggering systemic food-allergic reactions (Figure 1) [60].

Evidence from in vitro experiments supports the notion that food allergic sensitization occurs through the skin. In patients with peanut allergy, in vitro evaluation of the phenotype of peanut-specific CD4+ Th cells by using cutaneous lymphocyte antigen (CLA) as a marker for the epicutaneous rout of initial sensitization demonstrated that higher proportion of CD4+ Th cells expressed this skin-homing molecule (CLA+), indicating activation following exposure through the skin. The skin-homing CLA+ CD4+ Th cells revealed tendency toward Th2 polarization with increased production of IL-4 and IL-13 [65,66]. In experimental study Strid et al. found that epicutaneous sensitization after exposure to peanut protein through barrier-disrupted skin in BALB/c mice induced strong systemic Th2 immune response with highly elevated serum levels of IL-4 and peanut-specific IgE. Importantly, primary epicutaneous exposure inhibited the subsequent development of oral tolerance and also reduced existing tolerance to peanuts [67]. Furthermore, in murine models epicutaneous sensitization was associated with a significantly higher symptomatic response when compared with the oral, intragastric or intraperitoneal route of sensitization [68]. Kawasaki et al. demonstrated that BALB/c mice epicutaneously sensitized by regular exposition to OVA through disturbed skin exhibited higher serum concentration of OVA-specific IgE after oral challenge than intraperitoneally sensitized mice. Interestingly, in epicutaneously sensitized mice further skin barrier injury by tape-stripping augmented the allergic response to oral challenge even without additional topical antigen exposure [69].

The hypothesis of the progression from skin barrier dysfunction and epicutaneous food sensitization to the development of food allergy has been also confirmed in clinical studies. The first observation comes from the longitudinal birth cohort study (the ALSPAC Study) reporting an independent positive association of peanut allergy in preschool children with the cutaneous exposure to peanut allergens through the regular application of topical creams containing peanut oil on the inflamed skin. Most children who were allergic to peanuts had atopic dermatitis and had been topically exposed to peanut oil during the first 6 months of life [70]. Another study found the epidemiological link between significantly increased risk of IgE-mediated wheat allergy and current topical exposure to hydrolyzed wheat protein present in skin and hair care products [71]. In children with atopic dermatitis repeated application of creams containing oat was correlated with exacerbation of the skin lesions and higher rate of oat sensitization measured with oat-specific IgE, skin prick test or atopy patch test [72].

The development of epicutaneous food sensitization and subsequent food allergy may be also associated with high environmental exposure to food allergens [15]. Fox et al. showed that household peanut consumption, reflecting higher environmental exposure to peanut, increased the risk of peanut allergy among atopic infants with the greatest risk observed for peanut butter, which due to sticky nature may exert its impact through the skin [73]. It was further supported by strongly positive correlation that has been found between household peanut consumption and the concentration of peanut proteins in house dust, which additionally were able to activate mast cells and basophils in the skin [74]. The population-based birth cohort study indicated the dose-dependent association between early-life environmental exposure to peanut protein in household dust and subsequent development of peanut sensitization and peanut allergy in high risk infants and school-age children [75]. Importantly, this association was particularly observed in high-risk children with history of atopic dermatitis and its severe course and was strongly modified by the presence of null mutations in FLG gene, supporting the hypothesis of epicutaneous sensitization through impaired skin [76,77].

Important role of skin barrier impairment in epicutaneous food sensitization or food allergy has also been confirmed by studies objectively evaluating the function and integrity of this barrier by measuring transepidermal water loss (TEWL). Increased TEWL itself has been found to be strongly associated with food sensitization as assessed by skin prick testing in exclusively breastfed infants at 3 months of age [43]. Horimukai et al. reported that infants with increased TEWL in the first week of life have increased risk of subsequent development the sensitization to ovomucoid at age 32 weeks [78]. Another study in a large clinic-based cohort of children with atopic dermatitis revealed the relationship between allergic sensitization to peanuts and eggs and greater dysfunction of skin barrier defined as increased TEWL, even in non-lesional skin [79]. Leung et al. have performed a comprehensive evaluation of the stratum corneum with mulit-omnics approach and demonstrated that children with atopic dermatitis and food allergy presented unique skin abnormalities, such as increased TEWL, reduced FLG breakdown products and decrease proportion of ω-hydroxy fatty acid sphingosine ceramide content, which differentiates them from children without food allergy and nonatopic controls. Additionally, increased expression of keratins 5, 14, and 16 reflecting hyperproliferative keratinocytes, increased TEWL and decreased FLG breakdown products were identified as the most important predictors for atopic dermatitis with food allergy [80]. More recently, data from the population-based PreventADALL birth cohort confirmed that eczema, dry skin and raised TEWL at 3 months of age not only increased the risk of food sensitization at 6 months of age but also strongly predicted allergic sensitization from 3 to 6 months of age, with the highest positive predictive value reported for TEWL. The authors concluded that TEWL value higher than of 9.3 g/m^2^/h at 3 months in non-sensitized infants was the optimal prognostic marker to predict the development of food sensitization at 6 months of age [81].

## 4. Factors Affecting Skin Barrier Function

**Figure 2:**
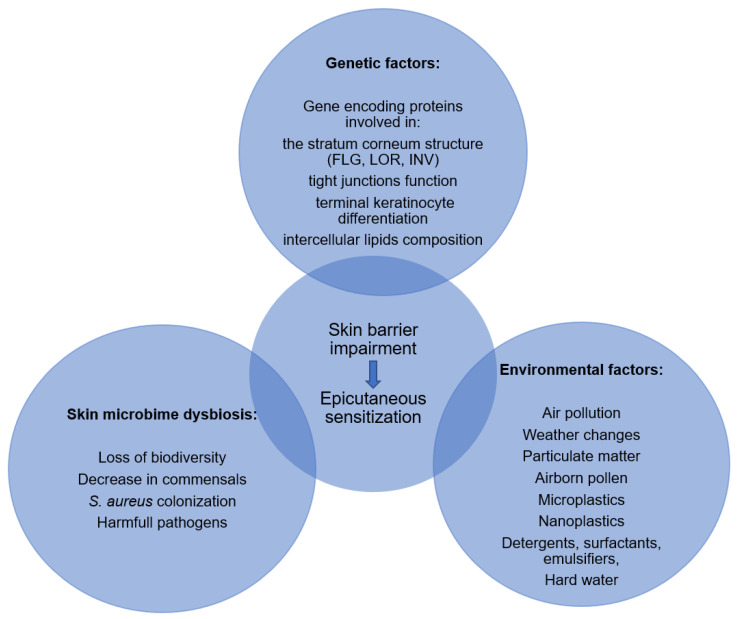
Factors causing skin barrier impairment and increasing the risk of epicutaneous sensitization.

Given that skin barrier impairment play a crucial role in the pathogenesis of atopic dermatitis and food allergy, it seems important to highlight the genetic and environmental factors that promote skin barrier dysfunction and thus increase the risk of epicutaneous sensitization (Figure 2).

### 4.1. Genetic Factors

Molecular genetics have clearly shown that disruption of skin barrier function can be attributed to genetic variants in genes encoding the structural proteins of the stratum corneum such as filaggrin (FLG), loricrin and involucrin [82,83,84]. Filaggrin is the most important epidermal structural protein that is crucial to maintaining stratum corneum function including keratinization, moisturization, preserving the epidermal barrier integrity and microbial defense [85]. In murine models FLG mutations directly contribute to changes in architecture of keratinocytes, abnormalities in lipid secretion, lower inflammatory thresholds for irritants and haptens, increased allergen penetration, and thus enhanced epicutaneous sensitization [85,86,87]. Data from clinical studies demonstrated that in FLG mutation were associated with atopic dermatitis-like phenotype, dry skin and impaired skin barrier function defined as lower level of natural moisturizing factors (NMF) and increased TEWL [87,88,89]. An impressive series of replication studies found FLG loss-of-function mutations to be the most significant genetic risk factor associated with atopic dermatitis, predisposing also to earlier onset, persistent course and more severe manifestation of the disease [90,91,92]. In recent years, FLG mutations have also been considered as risk factors for food sensitization and food allergy [93,94,95]. Data from cohorts of children from multiple countries showed a significant association of FLG mutations with challenge-proven peanut allergy, even in the absence of clinical evidence of atopic dermatitis [77,96,97]. Venkateraman et al. investigated the longitudinal correlation between FLG mutation, atopic dermatitis and the progression to food allergy over 18 years suggesting, based on path analysis, that FLG mutations are associated with an increased risk of atopic dermatitis in younger children and subsequent progression to food allergies in later life [46]. More recently, the large Genetics Of Food Allergy Study (GOFA) in children demonstrated that FLG mutations predispose to food allergy diagnosed by the DBPCFC to numerous foods including hen’s egg, cow’s milk, peanut, hazelnut, fish, soy, cashew, walnut, and sesame and in addition increased the risk of persistent course of allergy to hen’s egg and cow’s milk. Importantly, the association was independent of atopic dermatitis history and the allergenic food tested highlighting the role of genetic defects of epidermal barrier protein and skin barrier dysfunction as a common mechanism underlying all food allergies [98].

Beyond filaggrin, other mutations in genes encoding proteins involved in terminal keratinocyte differentiation, intercellular lipids composition and tight junctions function may contribute to cutaneous barrier dysfunction [39,50]. Loss-of-function mutations in SPINK5 gene result in defect in keratinocyte differentiation, increased kallikrein activity in the epidermis and enhanced skin barrier permeability [99]. Two independent murine studies found that mutation in the gene for mattrin (TMEM79), transmembrane protein involved in stratum corneum barrier function, alone lead to matted fur, atopic dermatitis-like lesions and elevated level of IgE [100,101]. Polymorphisms in the CLDN1 gene encoding claudin-1, tight junction transmembrane protein, was associated with atopic dermatitis and increased TEWL [102,103]. Recent GWASs have identified that other genes in the epidermal differentiation complex (EDC) such as hornerin, FLG2, involucrin and loricrin could impair skin barrier function in patients with atopic dermatitis, regardless of their FLG genotype [104,105,106]. Nevertheless, FLG deficiency appear to remain the most widely established risk factors for atopic dermatitis with the greatest impact on skin barrier structure and function. Importantly, growing evidence suggested that FLG expression can be downregulated by Th2-associated (IL-4, IL-13) and Th22-associated (IL-22) cytokines present in the skin during cutaneous inflammation [107,108]. The level of FLG expression is also modulated by environmental factors and exposomal influences [87,109].

### 4.2. Environmental Factors

Among environmental factors, air pollutions and weather changes have been shown to contribute in skin barrier dysfunction and result in the development as well as the exacerbation of atopic dermatitis [110]. The longitudinal study of the health of children in birth cohort with >17 years of follow-up reported that early exposure to oxidants such as O_3_ and NO_2_ at birth or/and in the first 3 years of life significantly increased the risk of developing atopic dermatitis [111]. In another observational study with atopic dermatitis patients changes in atmospheric parameters such as outdoor temperature, relative humidity and precipitation, increased concentration of particulate matter with aerodynamic diameter ≤10 μm (PM10), NO_2_ and O_3_, as well as raised total pollen counts were positively associated with skin reactivity and severity of atopic dermatitis symptoms [112]. The exacerbation of atopic dermatitis symptoms was also observed in children living in an industrial urban area after increased exposure to PM2.5 and PM10, with a stronger effect found on dry moderate days and for PM2.5 [113,114]. In vivo and in vitro studies have indicated that PM2.5 compromise skin barrier function by causing oxidative stress, DNA damage, downregulation of skin barrier-related protein expression and apoptosis through the mitochondria-regulated death pathway [115,116,117]. Recent experimental study provided evidence that PM2.5 induced FLG deficiency and subsequent skin barrier dysfunction in TNF-α, aryl hydrocarbon receptor (AHR) and TSLP dependent-manner. It was demonstrated that PM2.5 exposure inhibited FLG protein expression, increased TEWL and enhanced penetration of FITC-dextran in organotypic and mouse skin [118]. In recent years, microplastics and nanoplastics have attracted a lot of attention as the new environmental pollutants. Although the vast majority of studies focus on gastrointestinal and pulmonary toxicity, it has also been proposed that plastic particles can enter the skin through topical application of health and beauty products or through contact with water contaminated with nanoplastics and can damage the skin barrier by changing the expression of structural proteins and inducing the transcription of inflammatory genes [110,119,120].

Surfactants, detergents and cleansing products have also been proven as factors that even at trace concentration possess skin barrier-damaging properties, attributed to increasing skin pH, altered expression of keratinocytes differentiation markers, increased activity of epidermal proteases or reduction in their inhibition and in addition to promoting the release of pro-inflammatory cytokines [110,121]. Recent experimental study in mouse model showed that epicutaneous exposure to laundry detergents provoked skin barrier damage indicated by increased TEWL and in addition induced allergic skin inflammation manifested by greater expansion of eosinophils in the epidermis and local increase in production of IL-33, TSLP, IL-4 and IL-13 [122]. Data from clinical studies also demonstrated that anionic surfactants, particularly sodium lauryl sulfate (SLS) widely used in in cleansing products, cosmetics and detergents, affected the skin barrier integrity by both increasing TEWL and reducing stratum corneum hydration and also disrupting of tight junctions and interconnected molecules which enhances epidermal permeability [123,124]. Application of traditional alkaline soap on the skin resulted in elevated TEWL level and higher erythema index, which continued to increase even 24 and 72 h after exposure suggesting a prolonged effect of cleansers components on skin barrier damage [125]. Furthermore, higher frequency of using hand cleaning products and disinfectants during the COVID-19 pandemic was associated with exacerbation of symptoms in patients with atopic and contact dermatitis as well as with even with increased rate of new cases of these disease [126,127]. Another environmental factor that should be considered is so-called hard water containing high level of calcium and magnesium carbonates, as recently published meta-analysis has found positive correlation between domestic water hardness and prevalence of atopic dermatitis in school-age children [128]. It was also reported, that early life exposure to hard domestic water may contribute to an increased risk of development atopic dermatitis in infants [129,130]. Additionally, mechanistic study in humans suggested that exposure to hard water not only facilitates but also enhances deposition of surfactant sodium lauryl sulphate on the skin leading to impair functioning of epidermal barrier with increased TEWL, particularly in patients with atopic dermatitis carrying FLG mutations [131].

### 4.3. Skin Microbiome Dysbiosis

Skin microbiome research has highlighted that the microbial diversity with dominant commensals is essential to maintain cutaneous homeostasis while skin dysbiosis can cause significant skin barrier dysfunction and inappropriate immune response leading to epicutaneous sensitization [132,133]. In atopic dermatitis the composition of microbiota on the skin, both lesional and non-lesional, is altered with reduced commensal diversity and high prevalence of Staphylococcus species, in particular S. aureus colonization and secondary infection [134,135,136]. Moreover, the relative density of S. aureus is correlated with severity and exacerbation of atopic dermatitis suggesting an important role of S. aureus colonization in the disruption of the skin barrier [50,137,138]. S. aureus colonization has been shown to cause skin barrier dysfunction by negatively affecting skin hydration and stratum corneum integrity and permeability [139,140]. Significantly higher TEWL and skin pH was observed in atopic dermatitis patients colonized with S. aureus when compared to noncolonized patients and controls [139,141]. In addition, S. aureus promoted Th2-associated skin inflammation and allergic sensitization with elevated level of serum total IgE and peripheral blood eosinophil count [139,142]. Mechanisms by which S. aureus contributes to inflammation and skin barrier impairment involve decreasing expression of genes encoding tight junction protein and terminal differentiation markers, increasing protease and lipase activity and the production of exotoxins and proteins such as protein A and superantigens [134,140,142]. Experimental studies have demonstrated that concomitant skin exposures to peanut allergens and S. aureus superantigen, (Staphylococcal enterotoxin B (SEB) at doses observed in lesional skin lead to a significant enhancement of Th2-mediated immune response to peanuts extract and drive the development of peanut allergy [50,143]. In humans, metagenomic microbial analysis revealed increased abundance of S. aureus in non-lesional skin of patients with atopic dermatitis and food allergy, which was in addition positively correlated with elevated TEWL level [80]. Clinical study in children with atopic dermatitis showed the significant association between colonization with S. aureus and elevated level of sIgE for peanut, egg white, and cow’s milk as well as higher rate of allergic reaction after oral food challenge [144]. More recently data from LEAP study indicated that high-level sIgE production hen’s egg, peanut and cow’s milk allergen was related to S. aureus colonization independently on atopic dermatitis severity. Additionally, children colonized with S. aureus had a higher risk to develop persistent egg allergy suggesting that S. aureus can prevent the acquisition of natural tolerance to hen’s egg [145].

## 5. Epicutaneous Sensitization in the Pathogenesis of Food Allergy

Taking into account the important role of skin barrier dysfunction, the possible immune pathways associated with the progression from epicutaneous sensitization to food allergy have recently been studied in a murine models of food allergy and atopic dermatitis using tape stripping mouse skin as a model for scratching and mechanical injury [146,147,148]. It was proposed that crosstalk between skin and gut has its origin in mechanical skin damage which, by inducing epicutaneous food sensitization, can promote allergic reaction or even an anaphylaxis to foods leading to the enhancement of antigen-specific Th2 cytokine responses and the activation of IgE–mediated mast cell responses in the gastrointestinal tract [48]. *Bartinkas et al*. demonstrated that BALB/c mice epicutaneously sensitized by regular application of OVA on tape-stripped skin manifested systemic anaphylaxis following oral food challenge with IgE-dependent expansion of intestinal mast cells and increased production of IL-4 [147]. The existing evidence indicates that IL-33, IL-25 and TSLP induced by danger signals in tissues, are a key players in pathogenesis of epicutaneously induced food allergy 13,48]. This is also supported by the observation that all three alarmins are mandatory to induce food allergy in murine model, and once induced, only combined treatment with monoclonal antibodies blocking all of these cytokines leads to optimal suppression of murine food allergy [149].

IL-33 is released along with TSLP by keratinocytes in response to skin barrier damage and inflammation observed after mechanical injury and scratching or in atopic dermatitis [57]. Elevated levels of IL-33 are found in skin lesions and in serum of patients with atopic dermatitis, whereas the average concentration of IL-33 in serum can be up to 10 times higher than in healthy individuals [146,150,151]. In murine model, the level of circulating IL-33 increased 2-fold after mechanical injury affecting only 10% of the body surface area [152]. Further experimental studies in epicutaneously OVA-sensitized mice revealed that IL-33 released systemically after mechanical skin damage enlarges the intestinal mast cells load and stimulates IgE-mediated mast cell degranulation by ILC2 activation that produce IL-4 and IL-13 [146,148]. Thus, IL-33 may promote both food allergy symptoms in the intestine and food-induced anaphylaxis and conversely blocking of IL-33 signaling has been shown to inhibit anaphylactic reaction after oral challenge [146,148,153,154].

IL-25 along with IL-33 has been identified as primary cytokine inducing ILC2s in response to skin allergen exposure and in addition both IL-25 and IL-33 are required for activation of intestinal ILC2s and subsequent mast cells expansion [148,155]. It was demonstrated that intestinal mast cell accumulation failed after selective inactivation of IL-25 signaling [156]. The main source of IL-25 in the intestine are epithelial tuft cells whose number and ability to produce IL-25 significantly increase in the intestinal mucosa after skin mechanical injuries by tape stripping [148,155,157]. IL-25 drives the expansion of ILC2 in skin and small intestine and further mediates the secretion of IL-4 and IL-13, which in turn stimulates differentiation and activation of tuft cells causing a positive feedback loop [148,157,158]. Importantly, the IL-25 receptor was found to be preferentially expressed by intestinal ILC2, possibly explaining the exclusive accumulation of ILC2 in the small intestine after skin disruption [148]. A role for IL-25 in food allergy was proven in mice lacking IL-25 receptor that were more resistant to developing experimental IgE-mediated food allergy [158].

TSLP as another cytokine involved in skin-gut axis is produced by keratinocytes following skin barrier injury and cutaneous exposure to food allergens [148,159,160]. Overexpression of TSLP in skin keratinocytes and elevated level of TSLP in serum has been observed in patients with atopic dermatitis [161,162]. When released into the circulation, TSLP potentiate DC to preferentially differentiate naïve T cells to Th2 cells producing proinflammatory cytokines (IL-4, IL-5, IL-13) [163,164]. Furthermore, TSLP drives the recruitment of basophils in the skin-draining lymph nodes, stimulates their expansion in the skin and thus promotes basophil-dependent Th2 cytokine response in an IL-4–dependent manner [48]. Experimental model of food allergy in mice demonstrated that epicutaneous sensitization to OVA or peanut through an atopic dermatitis-like skin led to TSLP-dependent infiltration of basophils to the skin and basophil-derived IL-4 production that induce enhanced Th2 response in the skin [165]. Subsequently, intragastric OVA antigen challenge in these mice resulted in increased antigen-specific serum IgE synthesis and inflammation in the intestine with enhanced antigen-specific Th2 response, accumulation of intestinal mast cells and increased expression of mast cell–specific proteases which promotes the development of intestinal food allergy [166]. Suppression of the TSLP signaling and lack of basophil-derived IL-4 synthesis was found to inhibit food-induced allergic reaction in the intestine confirming the crucial role of TSLP-basophil-IL-4 axis in epicutaneous sensitization and associated pathogenesis of IgE-mediated food allergy [165,166].

Taken together, IL-33 released by keratinocytes acts jointly with IL-25 synthetized by tuft cells to stimulate expansion and activation of intestinal-resident ILC2 that produce IL-4 and IL-13. ILC2-derived IL-4 and IL-13 together with induced by TSLP basophil-derived IL-4 lead to enhanced expansion of activated mast cells and their IgE-mediated degranulation in the small intestine mucosa [48,156]. In turn, accumulated mast cells cause greater intestinal permeability, increased transepithelial passage and systemic absorption of food allergens leading enhanced sensitization to food in gastrointestinal tract and further allergic reaction or even an anaphylaxis to foods [13,48] (Figure 1).

## 6. Epicutaneous Sensitization in Unaffected Skin

Growing evidence implicating the skin may be an important site for the induction of allergic sensitization and subsequent allergic reaction prompting the question of whether the impairment of the skin barrier is an absolute prerequisite for the development of epicutaneous sensitization. Several studies have suggested that epicutaneous food sensitization may occur without skin barrier dysfunction, however the presence of an exogenous adjuvant or prolonged duration of exposure to the antigen was required for effective sensitization [167,168]. An experimental study in C3H/HeJ mice epicutaneously sensitized by topical application of the milk allergen α-lactalbumin to intact skin showed that antigen-specific IgE production was induced only in the presence of the pro-allergenic adjuvant (cholera toxin), as was not the case when exposed to the antigen alone [68]. In contrast, repeated topical exposure to peanut allergen extract was shown to be able to induce allergic sensitization in mice without additional adjuvants or skin damage possibly due to the inherent adjuvant activity of these allergens. Mice exposed to crude peanut extract through healthy skin exhibited increased serum level of IgE specific to the main peanut components Ara h 1 and Ara h 2 and anaphylactic reaction following intraperitoneal challenge. Additionally, peanut protein was postulated to caused bystander sensitization to milk allergen α-lactalbumin as concurrent application of peanut allergens and milk allergen α-lactalbumin on intact skin resulted in production of α-lactalbumin-specific IgE and anaphylaxis in mice orally challenged with milk allergen alone. All taken together may indicate that peanut allergens have an adjuvant effect when applied to the skin [169]. In humans, a significantly higher incidence of new cases of an immediate allergic reaction to wheat protein, and even anaphylaxis, has been described in previously unsensitized Japanese women after one year of regular daily use of facial soap containing hydrolyzed wheat protein on intact skin [170].

It is worth mentioning, that intact or clinically asymptomatic skin does not necessarily reflect the normal function of the skin barrier, which may be particularly the case in high-risk children such as FLG gene mutation carriers, those with atopic dermatitis or a family predisposition towards atopy. Numerous epidermal abnormalities have been demonstrated, at functional and molecular level, in nonlesional—that is, apparently healthy—skin of patients with atopic dermatitis [92,171,172]. The epidermal expression of TSLP, displaying response of keratinocytes to skin barrier damage, was found to be upregulated at as early as age 2 months in asymptomatic infants with family history of atopic dermatitis, correlating with higher risk of atopic dermatitis at 2 years of age [160]. Moreover, increased TEWL has been observed in the first weeks of life in infants carrying FLG mutations, even well before the development of atopic dermatitis symptoms, suggesting that skin barrier impairment precedes allergen sensitization and further clinical manifestation. Indeed, increased TEWL in these newborns predisposed to development of atopic dermatitis and food sensitization later in life [78,89].

## 7. Moisturizers Therapy as Topical Intervention to Improve Skin Barrier Function

Based on the aforementioned insights into the link between skin barrier impairment, epicutaneous sensitization, atopic dermatitis and food allergy, restoring skin barrier function to prevent or treat atopic dermatitis could be also a potential prevention strategy for related food allergy. Regarding barrier protection, moisturizers seem to be logic candidate especially since they are highly recommended integral part of atopic dermatitis management [173]. Appropriately applicated moisturizers restore skin barrier hydration, reduced skin dryness, relief itch and potentially have positive effect of skin barrier function [174]. If moisturizers can improve skin barrier function, they could be a simple and cost-effective prophylactic and therapeutic strategy.

### 7.1. Primary Prevention

The concept of using moisturizers for primary prevention of atopic dermatitis and food allergy is based on the evidence suggested that skin barrier dysfunction leading to allergic sensitization may occur in unaffected skin of high-risk children. In this regard, prophylactic targeting of the skin barrier should be introduced very early in life before the onset of clinical manifestation of atopic dermatitis. Early initiation of skin protection seems to be very important because the skin of neonates and infants is relatively immature and fully competent barrier function is formed only around 6 months of age [175,176,177].

The first two randomized, controlled pilot studies in high risk neonates demonstrated that daily application of standard petrolatum-based moisturizers on the whole body, started within first weeks after birth, were effective in preventing later incidence of atopic dermatitis with a relative risk reduction of 32% in Japanese population and 50% in Caucasian population [178,179]. In later studies, daily moisturizers therapy in infants reduced risk of dry skin and diaper dermatitis and this was associated with lowered TEWL, lowered pH and increased stratum corneum hydration providing evidence that the protective effect of moisturizers is due to improved skin function [180,181]. Similarly, decreasing trend in atopic dermatitis was observed by McClanahan et al. for moisturizers containing ceramide and amino acids in 2-year randomized controlled trial of high-risk newborns [182]. These promising studies were followed by large randomized, controlled trials that provided contradictory outcomes [183,184,185,186]. One of these studies, evaluating preventive therapy with emollients and synbiotics in healthy infants failed to confirm the protective effect of either emollients or synbiotics, used alone or in combination, on the development of atopic dermatitis and food allergy at 1 year of age [183]. Findings from the largest primary prevention study (the PrevetADALL) conducted in general population based cohort of 2397 infants did not indicate that the regular use of emollients baths and facial creams from 2 weeks of age significantly reduced the risk of atopic dermatitis by the age 1 year [184]. Recent analyses of the effect of emollients on the risk of food allergy in this cohort, when the children reach age 3 years, did not provide evidence that the application of petrolatum-based emollients from early infancy prevent the food allergy [187]. Another large, multicenter study (the BEEP) in infants at high risk of developing atopic dermatitis did not support the hypothesis that daily usage of regular emollients during infancy can delay, suppress or prevent atopic dermatitis at age 2 years. In the intervention group, the risk of food allergy was not reduced, however an increased rate of parent-reported skin infections was observed [185]. The five-year follow-up of infants enrolled in the BEEP study confirmed the lack of long-term clinical benefits for daily emollient application for the first year of life in preventing or delaying atopic dermatitis or food allergy [186]. Considering that the petrolatum-based emollients used in the above trials are now reported as less effective in restoring skin barrier function it is conceivable that different emollient formulation could provide a protective effect [176]. In the pilot study (the PEBBLES), regular application of trilipid ceramide-dominant emollient for the first 6 months of life showed decreasing trend in atopic dermatitis and food sensitization in high-risk infants at 6 and 12 months of age. Pre-protocol analysis found significantly lower rate of food sensitization at 12 months but only in infants treated with emollient twice daily at least 5 days a week [188]. Further follow-up is ongoing to validate these results and determine the long-term effectiveness of this next-generation emollient after cessation of treatment [189]. Another large ongoing trial (the CASCADE) aim to assess the efficacy of daily use of lipid-rich emollient in the general population recruited from a primary care setting [190]. In addition, recently published preliminary findings form pilot study in infant indicated that a trilipid cream may not only improve skin barrier integrity, as measured by TEWL, but also augment allergic sensitization or even induce tolerance which was reflected by lower level of total IgE and proinflammatory IL-4+ Tcells and increased level of tolerogenic IL-10 expressing T cells after 12 weeks treatment [191,192].

A number of emollient primary prevention studies are still ongoing and their results are awaited with great interest, especially as some research hinted at possible risk. The Cochrane systemic review and meta-analysis analyzed broad spectrum of skin interventions (including topical emollients and bath products) and determined that this skin care interventions during infancy are probably incapable to prevent atopic dermatitis and might even facilitate skin infections. Authors also concluded that data to assess the impact of emollients on the risk of food allergy were uncertain. What’s more, in conducted analysis of data from one trial that emollients applied during daily baths with paraffin-based bath oil may increase the risk of atopic dermatitis, indicating that daily bathing could potentially lead to decreasing of the skin barrier integrity [193]. This observation was supported by results from the EAT study demonstrating a correlation between daily bathing, particularly with bath oils, and elevated TEWL reflecting skin barrier impairment as well as higher rate of atopic dermatitis at 3 months [194]. More recently, post-hoc analyses of the EAT study reported that frequent using of moisturizers at 3 months of age was significantly associated with increased concurrent level of TEWL and the subsequent development of food allergy at 12 and 36 months. This relationship was dose-dependent as the risk of developing food allergy calculated as the odds ratio increased by 20% with each additional moisturization per week. Considering the reasons of reported association, authors postulated that moisturizers may damage the skin barrier as reflected in the dose-related correlation observed between increasing moisturization frequency and higher level of TEWL at 3 months of age. Moisturizers may also facilitate the penetration of the allergens across the skin the after their transferring from the hands of infants caregivers during the moisturizers application [195]. However, this alarming observation need to be confirmed, especially since moisturizers used in this study included mainly natural oils or mineral oils that have been reported to impede skin barrier function and have skin-penetration-enhancing properties [196,197,198].

In summary, primary prevention studies on atopic dermatitis and food allergy evaluating the efficacy of moisturizers provided mixed results that are insufficient to implement this strategy as primary prevention for the general population. However, two recently published meta-analysis presented promising results, particularly for atopic dermatitis, and justify the need for future research. Zhong et al. based on meta-analysis of aggregated-date from 10 studies concluded that prophylactic application of moisturizers from early infancy may prevent atopic dermatitis in high-risk children and only when moisturizers were used continuously. The authors hypothesized that moisturizers, by inhibiting the damaging effects of increased TEWL early in life, may delay rather than prevent the development of atopic dermatitis. Moisturizers intervention was not effective for preventing food allergy in this analysis [199]. Recent meta-analysis of 16 trial involving 5643 participants indicated for at high-risk newborns that emollients application from the neonatal period reduce the risk of atopic dermatitis and may have a little protective effect against food allergy. In case of healthy newborns analysis showed little or no preventive effect on development of atopic dermatitis whereas impact on risk of food allergy was uncertain [200].

### 7.2. Secondary Prevention

According to “outside-inside-outside” hypothesis, skin barrier disruption trigger the Th2 inflammation by upregulation of pro-inflammatory cytokines expression and conversely, the Th2 immunity cytokines can in turn impair skin barrier function by reducing expression of stratum corneum proteins and lead to enhanced epicutaneous allergen priming to form a vicious cycle [52,107,108,201]. It is also well known, that higher severity and more persistent course of atopic dermatitis is particularly associated with increased risk of developing food allergy [40]. Therefore, effective management of atopic dermatitis by both repairing the skin barrier function and proactive anti-inflammatory treatment could reduce epicouatnous sensitization and Th2 inflammation and potentially prevent development of food allergy. Experimental study in mice epicutaneously sensitized to OVA revealed that pretreatment with topical corticosteroids suppressed expansion of eosinophils in the skin and intestinal mucosa as well as allergic symptoms induced after oral challenge [69]. The retrospective cohort study of patients with moderate to severe atopic dermatitis indicated that patients who maintained proactive therapy with topical corticosteroids for 2 years had significantly decreased total serum IgE and food-specific IgE levels during the follow-up [202]. Recently, other hospital-based retrospective cohort study demonstrated that early treatment with emollients and topical corticosteroids in infants with atopic dermatitis not only shortened the duration of exacerbation but also contributed to lower rate of food allergy at age 2 years; the time period from the onset of symptoms until the start of aggressive treatment was a risk factor for later food allergies [203]. The randomized controlled trial—The Japanese Prevention of Allergy via Cutaneous Intervention (PACI)—is currently in progress to confirm these results [204].

### 7.3. Controversies and Future Challenges

In the light of the strongly suggestive data from molecular and experimental studies supporting the hypothesis that moisturizers therapy via repairing the skin barrier could prevent atopic dermatitis to thereby food allergy, the contradictory findings from further, large trials seem to be surprising and disappointing, especially after such promising results of preliminary studies. These unexpected and contrasting outcomes raises many questions and highlights numerous unmet needs.

The discrepancies in effect of moisturizers observed across studies can be explained by heterogeneity in recruited populations, sample size and adherence rate, criteria and timing of diagnosis and follow-up time as well as differences in interventions used. The infants included across the studies come from various geographical regions, have different genetic predispositions and were exposed to diverse environment factors. In addition, some trials recruited infants from the general population [183,184], while other studies selected only children at high-risk of atopic dermatitis [187,188]. This, in turn, leads to differences in the definition of “high-risk” population used and also raises the question how precisely determine whether the healthy newborns are not “at-risk”. Relatively small sample size in some studies, low rate of adherence with study protocol and contamination in the control group for which use of other moisturizers was allowed in most studies may account for disparity between the results. The heterogeneity in diagnostic criteria of atopic dermatitis and in particular food allergy may also affect the outcomes. Other and possibly key reason for contradictory results is differences in clinical study design including age of first intervention, duration, frequency and site of moisturizers application. Intervention duration varied between studies, ranging from 6 to 24 months, with one meta-analysis suggesting that only continuous use of emollients is effective [199]. Regarding the site of application, the spectrum of interventions used was also broad with bathing, whole body application or only to the face, making comparison difficult. Late age of first application, especially after sensitization has progressed, may not be effective in preventing food allergy. In addition, delayed start of proactive therapy in infants was a risk factor for food allergy [203]. Frequency of application ranged from 3–4 times a week, daily to twice daily, while in PEBBLES study emollients reduced the risk of food allergy only when applied twice daily at least 5 days a week [188]. There are still sparse data on the ideal frequency of emollients application required for best effectiveness. Although more frequent use would appear to be more effective, this approach may however lead to decreased adherence. Taken together, there is a critical need for large, well-designed, randomized, controlled clinical trials and broad population studies focusing on precisely defined participant phenotypes and following consistent study protocols.

The type of moisturizers used in above studies should be considered as the main reason for the discrepancies in the obtained results. Moisturizers vary widely in composition, pH, properties on skin hydration and effectiveness in repairing skin barrier function and integrity [174,198]. Since there is no single universal definition or standard classification of moisturizers, they consist of occlusive agents, emollients, humectants and lipid components in various combination [205]. Classic moisturizers, most commonly used in the aforementioned studies, are based on occlusive ingredients such as petrolatum and mineral oils which are able to reduce TEWL, but require frequent application and as inert agents do not address the biochemical abnormalities in the skin barrier [176,206]. Much more promising results were obtained with the use of a new generation of lipid-rich moisturizers containing ceramides, cholesterol and fatty acids that directly restore skin barrier function by replacing the intercellular lipids in the stratum corneum which are reduced in atopic dermatitis. Importantly, for optimal synergistic effect in restoring skin barrier function, these 3 lipids should be provided as a ceramide-dominant mixture in a physiological equimolar ratio (3:1:1) as is the case in the trilipid cream used in some studies [176,207]. Moisturizers containing emollients and humectants such as glycerol and urea found to be more effective when compared with emollient only preparation [208]. However, glycerol and urea formulations demonstrated complex effect on skin barrier function, strengthening skin barrier and accelerating repair after mechanical injury on one hand and on the other hand enhancing the permeability in higher concentration [209,210]. Among natural oils that were used in some studies as moisturizers, coconut oil and sunflower oil containing linoleic acid may provide some beneficial effects, but they also have the ability to disrupt the skin barrier, increase allergen penetration and induce sensitization, which is particularly evident with olives oil [211]. In addition to beneficial components, moisturizers may contain other excipients such as emulsifiers, preservatives, fragrances that may also have sensitizing or irritating potential [174,212]. Recently published systemic review, showed that the vast majority (75%) of moisturizers examined in clinical trials comprise at least one common contact allergen which also possess irritant potential and has been identified as a risk factor for contact dermatitis [213]. It is important in planned and future studies to use careful selection criteria for the type of moisturizers taking into account the optimal composition and formulation determining effectiveness and safety as well as patient preference as this intervention require high adherence rate to be effective. More head-to-head clinical trials will be required to compare the effectiveness of various moisturizers and establish whether the protective effect is limited to certain types of moisturizer or to specific population.

While accepting the hypothesis that skin barrier impairment precedes allergen sensitization and further clinical manifestation, an important future challenge is the development of minimally invasive skin sampling techniques allowing for early identification of skin barrier dysfunction and timely initiation of preventive strategies in neonates and infants. Currently available non-invasive clinical devices assessing TEWL, capacitance and skin pH as well as new methods such as Raman and impedance spectroscopy measure a broad spectrum of physiological traits of the epidermis but it remains to be elucidated exactly what biological abnormalities these readings reflect [49]. Future studies should also focus on identifying specific biomarkers to define various atopic dermatitis endotypes and to predict the risk of progression to food allergy which might allow the development of tailored preventive and therapeutic approaches.

**Figure 3:**
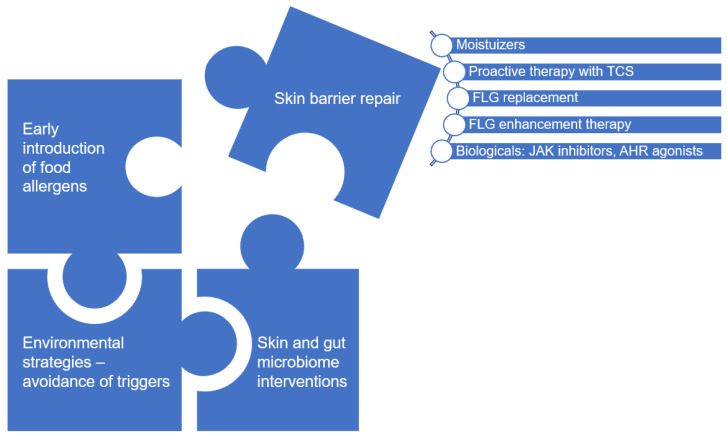
Possible directions for food allergy prevention and strategies targeting skin barrier repair. TCS—topical corticosteroids.

Contradictory results of reviewed studies highlight the complex pathogenesis of atopic dermatitis and food allergy, therefore targeting the skin barrier alone may not be a sufficiently effective preventive strategy. If the dual allergen exposure hypothesis is correct, then a combination of early allergenic food exposure and skin barrier improvement could be the optimal approach to reduce the risk of epicutaneous sensitization [187]. Considering the complex interplay between skin barrier dysfunction, immune dysregulation, microbiome dysbiosis and environmental factors it seems reasonable to hypothesize that all pieces of the puzzle need to be addressed to achieve the best clinical outcome (Figure 3). Many interventions have been studied in pregnant or breastfeeding women and infants to determine food allergy prevention strategies, including breastfeeding per se, diet diversity, dietary pattern, avoidance of food allergen, vitamin and mineral supplementation, fatty acid intake, fibers, prebiotic, probiotics and synbiotics, however, the results remain inconclusive in many cases [12,19]. Recently more attention has been paid to the gut and skin microbiome as a potential target for interventions against food allergy. Current evidence suggests that dysbiosis of the gut microbiome are not only linked to a higher risk of food allergy but also may be associated with the development of atopic dermatitis by affecting the skin immune system as well as skin microbiota [214,215,216]. On the other hand skin dysbiosis can cause significant skin barrier disruption leading to epicutaneous sensitization and development of food allergies [132,133,215]. Many strategies for modulating the microbiome are currently under investigation for the prevention of food allergy, including maternal Mediterranean diet in pregnancy and lactation, short-chain fatty acids (SCFAs), specifically butyrate and breastfeeding as one of the most important factors shaping the gut and skin microbiome in infants [110,217,218,219,220,221,222,223,224,225]. In addition, ongoing clinical trials are investigating the possibility of using the transfer of healthy skin microbiota or swabbing newborns with maternal vaginal microbiota as a new topical treatment for atopic dermatitis, which can be also a secondary prevention of food allergy [39,168,225]. Although dietary manipulation for normalization of the skin and gut microbiome dysbiosis, environmental control and treatment targeting Th2-cytokine inflammation are important preventive and management directions, since we focus on skin barrier-based interventions in this review, we have omitted their in-depth analysis from this overview.

Focusing on the skin barrier-based approach it should be emphasized that also in this case moisturizers probably represent one of many possible strategies, since the mechanisms causing skin barrier dysfunction are also varied. Therefore, other new pathogenesis-based topical interventions targeting skin barrier repair have been also investigated for treatment atopic dermatitis and thereby food allergy (Table 1).

## 8. New Topical Interventions Upregulating FLG Expression

Accepting the overwhelming evidence that FLG deficiency is a primary cause for the skin barrier abnormalities, restoring skin barrier function through upregulation of FLG expression could be beneficial strategies in both treating atopic dermatitis and preventing food allergy.

The most widely established cause for inherited FLG deficiency is the loss-of-function mutations of the FLG gene that lead to a reduction or complete loss of the FLG protein and its degradation products. Therefore, strategy aimed to treat this genetic defect or FLG replacement therapy is warranted. The potential gene-based approach to FLG replacement might include drugs that act on regulatory elements to control gene expression or “read-through” drugs that suppress a nonsense mutation and allow translation to proceed in the correct reading frame to produce a full-length FLG protein [226,227]. Although direct FLG gene-based therapy is currently not available, the development of “read-through” drugs for the treatment of atopic conditions has been patented. Another potentially attractive approach is direct FLG replacement via topical applications. In an experimental proof-of-concept study, Stout et al. gained encouraging results with the topical application of recombinant FLG monomer linked to a cell-penetrating peptide that was capable to penetrate epidermal tissue and after processing restore barrier function [228]. However, further studies are needed to establish safety and efficacy in humans. Indirect FLG replacement by topical application of its metabolites such as trans-urocanic acid and pyrrolidine carboxylic acid represents also an useful therapeutic approach, as FLG degradation products form the natural moisturizer factor (NMF) that is essential for maintenance the permeability barrier function [229]. Results from three phase 1/2b clinical trials confirmed the efficacy and safety of topical 5% cis-urocanic acid (cis-UCA) cream in treatment of patients with mild-to-moderate atopic dermatitis [230].

Additionally, it has been suggested that acquired mechanisms also modulate FLG expression, regardless of genetic mutations [105,247,248]. The FLG expression is downregulated in response to Th2-associated (IL-4, IL-13) and Th22-associated (IL-22) cytokines dysregulated in lesional and non-lesional skin of atopic dermatitis patients [201,249,250]. There are also findings available about external stimulants that may significantly affect FLG expression through different receptors and signaling pathways [40,87,248]. Therefore, strategies that involve blocking the cytokine-mediated FLG downregulation or enhancing FLG expression may be beneficial in treating atopic dermatitis.

The Janus kinase (JAK) inhibitors target the JAK-STAT signaling pathway which is involved in the regulation of multiple immune pathways implicated in atopic dermatitis pathogenesis, including Th2, Th22, Th1, and Th17 [251,252]. Since Th2 cytokines impact keratinocyte differentiation and hamper the expression of FLG through JAK-STAT signaling, JAK inhibitors potently upregulate FLG expression, restore skin barrier function and reduce skin inflammation [253,254]. In experimental studies, topical application of pan-JAK inhibitor, JTE-052, diminished TEWL level and increased the production of FLG and NMF in a murine model and human skin graft model of atopic dermatitis [231,255,256]. The topical form of JAK-kinase inhibitors, delgocitinib (JTE-052) and ruxolitinib, demonstrated remarkable improvement in disease severity and itch scores in several clinical trials and are currently approved for the treatment of atopic dermatitis [231,232,233,234,235].

Tapinarof and other high-affinity AHR (the aryl hydrocarbon receptor) agonists are currently being studied as promising candidates for new topical treatment of atopic dermatitis by enhancing FLG expression. Activation of AHR, which is transcription factor highly expressed in keratinocyte, induces upregulation of FLG and other proteins related to epidermal terminal differentiation and thus promotes skin barrier repair [257,258]. In clinical trials, phase 2/2b, tapinarof 1% cream, a naturally derived hydroxylated stilbene, showed clinically significant improvements in efficacy analyses, including severity scores (EASI, SCORAD, BSA, IGA), pruritus score and Patient Oriented Eczema Measure (POEM) scores [236,237]. Currently, three large phase 3 clinical trials for tapinarof are underway. Topically applied tryptophan photoproduct (FICH) upregulated FLG expression, improved clinical scores and reduced TEWL in murine dermatitis model [238,239]. Diosmin, another natural-derived AHR ligand, increased FLG expression and epidermal thickness as well as was able to reverse the Th2 cytokine-mediated FLG downregulation in normal human epidermal keratinocytes, thus it has been suggested to be the most promising phytochemical for atopic dermatitis treatment [240].

Many other molecules with FLG-enhancing properties have been considered as potential treating agents for atopic dermatitis. All investigated topical PPARs (the peroxisome proliferator-activated receptors) and LXR (the liver X receptor) agonists have been demonstrated to promote FLG expression, stimulate keratinocytes differentiation and reduce epidermal hyperplasia in mice and human epidermis models of atopic dermatitis [241,242]. Sirtuin 1 (SIRT1), a members of the class III histone deacetylase family, is another potential target as FLG has been found to be expressed in SIRT1-dependent manner [243]. Treatment with adiponectin increased SIRT1 expression leading to upregulation of FLG expression in human keratinocytes and thereby improving skin barrier permeability [244]. Several bioflavonoid compounds, such as apigenin, hesperidin and apigetrin, has also been shown to enhance FLG expression and significantly strengthen skin barrier integrity both in vivo and in vitro [245,246].

Although novel topical interventions enhancing FLG expression or blocking acquired FLG downregulation appear to be very promising, further studied are required to fully assess their efficacy and safety as well as to determine the beneficial effect in human for the few more that still have not advanced beyond in vitro and in vivo models.

## 9. Conclusions

In recent years, many previously unknown details regarding the mechanisms of food allergy pathogenesis have been clarified, pointing to a strong relationship between the impaired skin barrier and the development of food allergy, which is theoretically causal, through epicutaneous sensitization. Thus, the prophylactic and therapeutic interventions to restore skin barrier function are widely studied as a promising food allergy prevention strategy. It seems reasonable to assume that early intervention targeting the skin to prevent or modify the course of atopic dermatitis could potentially prevent the development of food allergy by reducing the risk of epicutanous sensitization. In this review we focused on topical skin barrier-strengthening interventions, mainly moisturizers, as a simple, achievable and safe prevention strategy. However, responses in clinical trials are varying depending on moisturizers components and formulation, frequency, duration and age of application indicating the need for a more precise approach in the future studies. In summary, some moisturizers, especially a complex, ceramide-based ones, are expected to prevent or delay the development of atopic dermatitis and food allergy in high-risk populations, whereas others—petrolatum-based—have limited effect. The evidence on the effect of moisturizers for food allergy prevention is very limited and uncertain, mainly due to the considerable difficulties in measuring food allergy outcomes in preventive studies. Nevertheless, the currently available data offer an exciting prospect for the future, however their heterogeneity does not allow to formulate definite recommendations at this time. Inconsistent results suggest that there is probably no “one-size-fits-all” approach to prevention and much remains to learned before presented strategies can be routinely implemented. We believe the future is very bright, however, there is still a need to design extensive mechanistic and clinical studies to fully elucidate the mechanism behind epicutaneous sensitization and the pathogenesis of food allergy. Based on the promising results of clinical trials, there is also reason to hope that novel topical interventions enhancing FLG expression or blocking acquired FLG downregulation, specifically the JAK-kinase inhibitors, will be available treatment and prevention options the near future. The key challenge now is to continue research into skin barrier-based interventions as well as to further search for other potential targets for the prevention of food allergies. The future findings, whether positive or negative, will further improve our understanding of the complexity of food allergy pathogenesis and bring us closer to the development of effective prevention strategies.

## Figures and Tables

**Table 1:** Topical interventions targeting skin barrier repair for prevention of atopic dermatitis and food allergy. TCS—topical corticosteroids.

Intervention	Area of Focus	References
Primary prevention Topical moisturizers		
	Preventive therapy with petrolatum-based moisturizers for atopic dermatitis Preventive therapy with moisturizers containing ceramide and amino acids for atopic dermatitis Preventive therapy with moisturizers and synbiotics for atopic dermatitis and food allergy Preventive therapy with petrolatum-based moisturizers for atopic dermatitis and food allergy Preventive therapy with trilipid ceramide-dominant moisturizers for atopic dermatitis and food allergy Link between moisturizers and food allergy	[178,179,180,181] [182] [183] [184,185,186,187] [188,189,190] [194]
Secondary prevention		
	Proactive atopic dermatitis therapy with TCS and moisturizers and the prevention of food allergy	[202,203,204]
Topical interventions upregulating FLG expression		
Gene-based approach Direct replacement of FLG Indirect replacement therapy JAK inhibitors Enhancement FLG expression	“Read-through” drugs Topical application of recombinant FLG monomer Topical application of FLG metabolites: trans-urocanic acid and pyrrolidine carboxylic acid Inhibition of cytokine-mediated FLG downregulation: delgocitinib (JTE-052) and ruxolitinib AHR agonists: tapinarof, tryptophan photoproduct (FICH), diosmin Peroxisome proliferator-activated receptors (PPARs) agonists Liver X receptor (LXR) agonists Sirtuin 1 (SIRT1) Bioflavonoids: apigenin, hesperidin, apigetrin	[226,227] [228] [229,230] [231,232,233,234,235] [236,237,238,239,240] [241] [242] [243,244] [245,246]
Microbiome interventions		
	Microbial transplantation of commensal bacteria	[225]

## Data Availability

Not applicable.

## References

[1] Sicherer S.H., Sampson H.A. (2018). Food allergy: A review and update on epidemiology, pathogenesis, diagnosis, prevention, and management. J. Allergy Clin. Immunol..

[2] Warren C.M., Jiang J., Gupta R.S. (2020). Epidemiology and burden of food allergy. Curr. Allergy Asthma. Rep..

[3] Lyons S.A., Clausen M., Knulst A.C., Ballmer-Weber B.K., Fernandez-Rivas M., Barreales L., Bieli C., Dubakiene R., Fernandez-Perez C., Jedrzejczak-Czechowicz M. (2020). Prevalence of food sensitization and food allergy in children across Europe. J. Allergy Clin. Immunol. Pract..

[4] Gupta R., Warren C., Smith B., Blumenstock J.A., Jiang J., Davis M.M., Nadeau K.C. (2018). The public health impact of parent-reported childhood food allergies in the United States. Pediatrics.

[5] Upton J., Alvaro M., Nadeau K. (2019). A perspective on the pediatric death from oral food challenge reported from the Allergy Vigilance Network. Allergy.

[6] Shaker M.S., Schwartz J., Ferguson M. (2017). An update on the impact of food allergy on anxiety and quality of life. Curr. Opin. Pediatr..

[7] Gupta R., Holdford D., Bilaver L., Dyer A., Holl J.L., Meltzer D. (2013). The Economic Impact of Childhood Food Allergy in the United States. JAM. A Pediatr..

[8] Scott L.A., Jones B.I., Berni T.R., Berni E.R., De Vries J., Currie C.J. (2019). Evaluation of the epidemiology of peanut allergy in the United Kingdom. Expert Rev. Clin. Immunol..

[9] Johansson E., Mersha T.B. (2021). Genetics of Food Allergy. Immunol. Allergy Clin. North Am..

[10] Davis E.C., Jackson C.M., Ting T., Harizaj A., Järvinen K.M. (2022). Predictors and biomarkers of food allergy and sensitization in early childhood. Ann. Allergy Asthma Immunol..

[11] Peters R.L., Mavoa S., Koplin J.J. (2022). An Overview of Environmental Risk Factors for Food Allergy. Int. J. Env. Res. Public Health.

[12] Brough H.A., Lanser B.J., Sindher S.B., Teng J.M.C., Leung D.Y.M., Venter C., Chan S.M., Santos A.F., Bahnson H.T., Guttman-Yassky E. (2022). Early intervention and prevention of allergic diseases. Allergy.

[13] Sampson H.A., O’Mahony L., Burks A.W., Plaut M., Lack G., Akdis C.A. (2018). Mechanisms of food allergy. J. Allergy Clin. Immunol..

[14] Peters R.L., Neeland M.R., Allen K.J. (2017). Primary Prevention of Food Allergy. Curr. Allergy Asthma Rep..

[15] Brough H.A., Nadeau K.C., Sindher S.B., Alkotob S.S., Chan S., Bahnson H.T., Leung D.Y.M., Lack G. (2020). Epicutaneous sensitization in the development of food allergy: What is the evidence and how can this be prevented?. Allergy.

[16] Lack G. (2008). Epidemiologic risks for food allergy. J. Allergy Clin. Immunol..

[17] Lack G. (2012). Update on risk factors for food allergy. J. Allergy Clin. Immunol..

[18] Nowak-Wegrzyn A., Szajewska H., Lack G. (2017). Food allergy and the gut. Nat. Rev. Gastroenterol. Hepatol..

[19] de Silva D., Halken S., Singh C., Muraro A., Angier E., Arasi S., Arshad H., Beyer K., Boyle R., du Toit G. (2020). European Academy of Allergy, Clinical Immunology Food Allergy, Anaphylaxis Guidelines Group. Preventing food allergy in infancy and childhood: Systematic review of randomised controlled trials. Pediatr. Allergy Immunol..

[20] Pabst O., Mowat A.M. (2012). Oral tolerance to food protein. Mucosal. Immunol..

[21] Garcia-Larsen V., Ierodiakonou D., Jarrold K., Cunha S., Chivinge J., Robinson Z., Geoghegan N., Ruparelia A., Devani P., Trivella M. (2018). Diet during pregnancy and infancy and risk of allergic or autoimmune disease: A systematic review and meta-analysis. PLoS Med..

[22] Perkin M.R., Logan K., Tseng A., Raji B., Ayis S., Peacock J., Brough H., Marrs T., Radulovic S., Craven J. (2016). Randomized Trial of Introduction of Allergenic Foods in Breast-Fed Infants. N. Engl. J. Med..

[23] Obbagy J.E., English L.K., Wong Y.P., Butte N.F., Dewey K.G., Fleischer D.M., Fox M.K., Greer F.R., Krebs N.F., Scanlon K.S. (2019). Complementary feeding and food allergy, atopic dermatitis/eczema, asthma, and allergic rhinitis: A systematic review. Am. J. Clin. Nutr.

[24] Burgess J.A., Dharmage S.C., Allen K., Koplin J., Garcia-Larsen V., Boyle R., Waidyatillake N., Lodge C.J. (2019). Age at introduction to complementary solid food and food allergy and sensitization: A systematic review and meta-analysis. Clin. Exp. Allergy.

[25] Trogen B., Jacobs S., Nowak-Wegrzyn A. (2022). Early Introduction of Allergenic Foods and the Prevention of Food Allergy. Nutrients.

[26] Feeney M., Du Toit G., Roberts G., Sayre P.H., Lawson K., Bahnson H.T., Sever M.L., Radulovic S., Plaut M., Lack G. (2016). Impact of peanut consumption in the LEAP Study: Feasibility, growth, and nutrition. J. Allergy Clin. Immunol..

[27] du Toit G., Sayre P.H., Roberts G., Lawson K., Sever M.L., Bahnson H.T., Fisher H.R., Feeney M., Radulovic S., Basting M. (2018). Allergen specificity of early peanut consumption and effect on development of allergic disease in the Learning Early About Peanut Allergy study cohort. J. Allergy Clin. Immunol..

[28] Natsume O., Kabashima S., Nakazato J., Yamamoto-Hanada K., Narita M., Kondo M., Saito M., Kishino A., Takimoto T., Inoue E. (2017). Two-step egg introduction for prevention of egg allergy in high-risk infants with eczema (PETIT): A randomised, double-blind, placebo-controlled trial. Lancet.

[29] Palmer D.J., Metcalfe J., Makrides M., Gold M.S., Quinn P., West C.E., Loh R., Prescott S.L. (2013). Early regular egg exposure in infants with eczema: A randomized controlled trial. J. Allergy Clin. Immunol..

[30] Wei-Liang Tan J., Valerio C., Barnes E.H., Turner P.J., Van Asperen P.A., Kakakios A.M., Campbell D.E., Beating Egg Allergy Trial (BEAT) Study Group (2017). A randomized trial of egg introduction from 4 months of age in infants at risk for egg allergy. J. Allergy Clin. Immunol..

[31] Bellach J., Schwarz V., Ahrens B., Trendelenburg V., Aksünger Ö., Kalb B., Niggemann B., Keil T., Beyer K. (2017). Randomized placebo-controlled trial of hen’s egg consumption for primary prevention in infants. J. Allergy Clin. Immunol..

[32] Peters R.L., Koplin J.J., Dharmage S.C., Tang M.L.K., McWilliam V.L., Gurrin L.C., Neeland M.R., Lowe A.J., Ponsonby A.L., Allen K.J. (2019). Early Exposure to Cow’s Milk Protein Is Associated with a Reduced Risk of Cow’s Milk Allergic Outcomes. J. Allergy Clin. Immunol. Pract..

[33] Urashima M., Mezawa H., Okuyama M., Urashima T., Hirano D., Gocho N., Tachimoto H. (2019). Primary Prevention of Cow’s Milk Sensitization and Food Allergy by Avoiding Supplementation With Cow’s Milk Formula at Birth: A Randomized Clinical Trial. JAMA Pediatr..

[34] Fisher H.R., Du Toit G., Bahnson H.T., Lack G. (2018). The challenges of preventing food allergy: Lessons learned from LEAP and EAT. Ann. Allergy Asthma Immunol..

[35] Voorheis P., Bell S., Cornelsen L., Quaife M., Logan K., Marrs T., Radulovic S., Craven J., Flohr C., Lack G. (2019). Challenges experienced with early introduction and sustained consumption of allergenic foods in the Enquiring About Tolerance (EAT) study: A qualitative analysis. J. Allergy Clin. Immunol..

[36] Sakihara T., Otsuji K., Arakaki Y., Hamada K., Sugiura S., Ito K. (2022). Early Discontinuation of Cow’s Milk Protein Ingestion Is Associated with the Development of Cow’s Milk Allergy. J. Allergy Clin. Immunol. Pract..

[37] Keet C., Pistiner M., Plesa M., Szelag D., Shreffler W., Wood R., Dunlop J., Peng R., Dantzer J., Togias A. (2021). Age and eczema severity, but not family history, are major risk factors for peanut allergy in infancy. J. Allergy Clin. Immunol..

[38] Walker M.T., Green J.E., Ferrie R.P., Queener A.M., Kaplan M.H., Cook-Mills J.M. (2018). Mechanism for initiation of food allergy: Dependence on skin barrier mutations and environmental allergen costimulation. J. Allergy Clin. Immunol..

[39] Cook-Mills J.M., Emmerson L.N. (2022). Epithelial barrier regulation, antigen sampling, and food allergy. J. Allergy Clin. Immunol..

[40] Tsakok T., Marrs T., Mohsin M., Baron S., du Toit G., Till S., Flohr C. (2016). Does atopic dermatitis cause food allergy? A systematic review. J. Allergy Clin. Immunol..

[41] Perkin M.R., Logan K., Bahnson H.T., Marrs T., Radulovic S., Craven J., Flohr C., Mills E.N., Versteeg S.A., van Ree R. (2019). Efficacy of the Enquiring About Tolerance (EAT) study among infants at high risk of developing food allergy. J. Allergy Clin. Immunol..

[42] Du Toit G., Roberts G., Sayre P.H., Plaut M., Bahnson H.T., Mitchell H., Radulovic S., Chan S., Fox A., Turcanu V. (2013). Identifying infants at high risk of peanut allergy: The Learning Early About Peanut Allergy (LEAP) screening study. J. Allergy Clin. Immunol..

[43] Flohr C., Perkin M., Logan K., Marrs T., Radulovic S., Campbell L.E., MacCallum S.F., McLean W.H.I., Lack G. (2014). Atopic dermatitis and disease severity are the main risk factors for food sensitization in exclusively breastfed infants. J. Investig. Dermatol..

[44] Martin P.E., Eckert J.K., Koplin J.J., Lowe A.J., Gurrin L.C., Dharmage S.C., Vuillermin P., Tang M.L., Ponsonby A.L., Matheson M. (2015). HealthNuts Study Investigators. Which infants with eczema are at risk of food allergy? Results from a population-based cohort. Clin. Exp. Allergy.

[45] Eller E., Kjaer H.F., Høst A., Andersen K.E., Bindslev-Jensen C. (2009). Food allergy and food sensitization in early childhood: Results from the DARC cohort. Allergy.

[46] Venkataraman D., Soto-Ramírez N., Kurukulaaratchy R.J., Holloway J.W., Karmaus W., Ewart S.L., Arshad S.H., Erlewyn-Lajeunesse M. (2014). Filaggrin loss-of-function mutations are associated with food allergy in childhood and adolescence. J. Allergy Clin. Immunol..

[47] Lowe A.J., Abramson M.J., Hosking C.S., Carlin J.B., Bennett C.M., Dharmage S.C., Hill D.J. (2007). The temporal sequence of allergic sensitization and onset of infantile eczema. Clin. Exp. Allergy.

[48] van Splunter M., Liu L., van Neerven R.J.J., Wichers H.J., Hettinga K.A., de Jong N.W. (2020). Mechanisms Underlying the Skin-Gut Cross Talk in the Development of IgE-Mediated Food Allergy. Nutrients.

[49] Yoshida T., Beck L.A., De Benedetto A. (2022). Skin barrier defects in atopic dermatitis: From old idea to new opportunity. Allergol. Int..

[50] Leung D.Y.M., Berdyshev E., Goleva E. (2020). Cutaneous barrier dysfunction in allergic diseases. J. Allergy Clin. Immunol..

[51] Wickett R.R., Visscher M.O. (2009). Structure and function of the epidermal barrier. Am. J. Infect. Control.

[52] Elias P.M. (2008). Skin barrier function. Curr. Allergy Asthma Rep..

[53] Darlenski R., Kazandjieva J., Tsankov N. (2011). Skin barrier function: Morphological basis and regulatory mechanisms. J. Clin. Med..

[54] Lefèvre-Utile A., Braun C., Haftek M., Aubin F. (2021). Five Functional Aspects of the Epidermal Barrier. Int. J. Mol. Sci.

[55] Eyerich S., Eyerich K., Traidl-Hoffmann C., Biedermann T. (2018). Cutaneous Barriers and Skin Immunity: Differentiating A Connected Network. Trends Immunol..

[56] Lee S.H., Jeong S.K., Ahn S.K. (2006). An update of the defensive barrier function of skin. Yonsei Med. J..

[57] Hammad H., Lambrecht B.N. (2015). Barrier Epithelial Cells and the Control of Type 2 Immunity. Immunity.

[58] Werfel T., Allam J.P., Biedermann T., Eyerich K., Gilles S., Guttman-Yassky E., Hoetzenecker W., Knol E., Simon H.U., Wollenberg A. (2016). Cellular and molecular immunologic mechanisms in patients with atopic dermatitis. J. Allergy Clin. Immunol..

[59] Humeniuk P., Dubiela P., Hoffmann-Sommergruber K. (2017). Dendritic cells and their role in allergy: Uptake, proteolytic processing and presentation of allergens. Int. J. Mol. Sci..

[60] Palomares O., Akdis M., Martín-Fontecha M., Akdis C.A. (2017). Mechanisms of immune regulation in allergic diseases: The role of regulatory T and B cells. Immunol. Rev..

[61] Pasha M.A., Patel G., Hopp R., Yang Q. (2019). Role of innate lymphoid cells in allergic diseases. Allergy Asthma Proc..

[62] Zheng H., Zhang Y., Pan J., Liu N., Qin Y., Qiu L., Liu M., Wang T. (2021). The Role of Type 2 Innate Lymphoid Cells in Allergic Diseases. Front. Immunol..

[63] Satitsuksanoa P., Daanje M., Akdis M., Boyd S.D., van de Veen W. (2021). Biology and dynamics of B cells in the context of IgE-mediated food allergy. Allergy.

[64] Tordesillas L., Berin M.C., Sampson H.A. (2017). Immunology of Food Allergy. Immunity.

[65] Chan S.M., Turcanu V., Stephens A.C., Fox A.T., Grieve A.P., Lack G. (2012). Cutaneous lymphocyte antigen and alpha4beta7 T-lymphocyte responses are associated with peanut allergy and tolerance in children. Allergy.

[66] Weissler K.A., Rasooly M., DiMaggio T., Bolan H., Cantave D., Martino D., Neeland M.R., Tang M.L.K., Dang T.D., Allen K.J. (2018). Identification and analysis of peanut-specific effector T and regulatory T cells in children allergic and tolerant to peanut. J. Allergy Clin. Immunol..

[67] Strid J., Strobel S. (2005). Skin barrier dysfunction and systemic sensitization to allergens through the skin. Curr. Drug Targets-Inflamm. Allergy.

[68] Dunkin D., Berin M.C., Mayer L. (2011). Allergic sensitization can be induced via multiple physiologic routes in an adjuvant-dependent manner. J. Allergy Clin. Immunol..

[69] Kawasaki A., Ito N., Murai H., Yasutomi M., Naiki H., Ohshima Y. (2018). Skin inflammation exacerbates food allergy symptoms in epicutaneously sensitized mice. Allergy.

[70] Lack G., Fox D., Northstone K., Golding J., Avon Longitudinal Study of Parents and Children Study Team (2003). Factors associated with the development of peanut allergy in childhood. N. Engl. J. Med..

[71] Fukutomi Y., Taniguchi M., Nakamura H., Akiyama K. (2014). Epidemiological link between wheat allergy and exposure to hydrolyzed wheat protein in facial soap. Allergy.

[72] Boussault P., Léauté-Labrèze C., Saubusse E., Maurice-Tison S., Perromat M., Roul S., Sarrat A., Taïeb A., Boralevi F. (2007). Oat sensitization in children with atopic dermatitis: Prevalence, risks and associated factors. Allergy.

[73] Fox A.T., Sasieni P., du Toit G., Syed H., Lack G. (2009). Household peanut consumption as a risk factor for the development of peanut allergy. J. Allergy Clin. Immunol..

[74] Brough H.A., Santos A.F., Makinson K., Penagos M., Stephens A.C., Douiri A., Fox A.T., Du Toit G., Turcanu V., Lack G. (2013). Peanut protein in household dust is related to household peanut consumption and is biologically active. J. Allergy Clin. Immunol..

[75] Brough H.A., Kull I., Richards K., Hallner E., Söderhäll C., Douiri A., Penagos M., Melén E., Bergström A., Turcanu V. (2018). Environmental peanut exposure increases the risk of peanut sensitization in high-risk children. Clin. Exp. Allergy.

[76] Brough H.A., Simpson A., Makinson K., Hankinson J., Brown S., Douiri A., Belgrave D.C., Penagos M., Stephens A.C., McLean W.H. (2014). Peanut allergy: Effect of environmental peanut exposure in children with filaggrin loss-of-function mutations. J. Allergy Clin. Immunol..

[77] Brough H.A., Liu A.H., Sicherer S., Makinson K., Douiri A., Brown S.J., Stephens A.C., Irwin McLean W.H., Turcanu V., Wood R.A. (2015). Atopic dermatitis increases the effect of exposure to peanut antigen in dust on peanut sensitization and likely peanut allergy. J. Allergy Clin. Immunol..

[78] Horimukai K., Morita K., Narita M., Kondo M., Kabashima S., Inoue E., Sasaki T., Niizeki H., Saito H., Matsumoto K. (2016). Transepidermal water loss measurement during infancy can predict the subsequent development of atopic dermatitis regardless of filaggrin mutations. Allergol. Int..

[79] Sherenian M.G., Kothari A., Biagini J.M., Kroner J.W., Baatyrbek Kyzy A., Johannson E., Atluri G., He H., Martin L.J., Khurana Hershey G.K. (2021). Sensitization to peanut, egg or pets is associated with skin barrier dysfunction in children with atopic dermatitis. Clin. Exp. Allergy.

[80] Leung D.Y.M., Calatroni A., Zaramela L.S., LeBeau P.K., Dyjack N., Brar K., David G., Johnson K., Leung S., Ramirez-Gama M. (2019). The nonlesional skin surface distinguishes atopic dermatitis with food allergy as a unique endotype. Sci. Transl. Med..

[81] Wärnberg Gerdin S., Lie A., Asarnoj A., Borres M.P., Lødrup Carlsen K.C., Färdig M., Konradsen J.R., Monceyron Jonassen C., Olsson Mägi C.A., Rehbinder E.M. (2022). Impaired skin barrier and allergic sensitization in early infancy. Allergy.

[82] Elias P.M. (2018). Primary role of barrier dysfunction in the pathogenesis of atopic dermatitis. Exp. Dermatol..

[83] Martin M.J., Estravís M., García-Sánchez A., Dávila I., Isidoro-García M., Sanz C. (2020). Genetics and Epigenetics of Atopic Dermatitis: An Updated Systematic Review. Genes.

[84] Ferreira M.A.R., Vonk J.M., Baurecht H., Marenholz I., Tian C., Hoffman J.D., Helmer Q., Tillander A., Ullemar V., Lu Y. (2019). Eleven loci with new reproducible genetic associations with allergic disease risk. J. Allergy Clin. Immunol..

[85] Moosbrugger-Martinz V., Leprince C., Méchin M.-C., Simon M., Blunder S., Gruber R., Dubrac S. (2022). Revisiting the Roles of Filaggrin in Atopic Dermatitis. Int. J. Mol. Sci..

[86] Elias M.S., Long H.A., Newman C.F., Wilson P.A., West A., McGill P.J., Wu K.C., Donaldson M.J., Reynolds N.J. (2017). Proteomic analysis of filaggrin deficiency identifies molecular signatures characteristic of atopic eczema. J. Allergy Clin. Immunol..

[87] Drislane C., Irvine A.D. (2020). The role of filaggrin in atopic dermatitis and allergic disease. Ann. Allergy Asthma Immunol..

[88] Kezic S., Kemperman P.M., Koster E.S., de Jongh C.M., Thio H.B., Campbell L.E., Irvine A.D., McLean W.H., Puppels G.J., Caspers P.J. (2008). Loss-of-function mutations in the filaggrin gene lead to reduced level of natural moisturizing factor in the stratum corneum. J. Investig. Dermatol..

[89] Flohr C., England K., Radulovic S., McLean W.H., Campbel L.E., Barker J., Perkin M., Lack G. (2010). Filaggrin loss-of-function mutations are associated with early-onset eczema, eczema severity and transepidermal water loss at 3 months of age. Br. J. Dermatol..

[90] Tenn M.W., Ellis A.K. (2016). The clinical relevance of filaggrin mutations: Effect on allergic disease. Ann. Allergy Asthma Immunol..

[91] Gupta J., Margolis D.J. (2020). Filaggrin gene mutations with special reference to atopic dermatitis. Curr. Treat. Options Allergy.

[92] Langan S.M., Irvine A.D., Weidinger S. (2020). Atopic dermatitis. Lancet.

[93] Carter C.A., Frischmeyer-Guerrerio P.A. (2018). The Genetics of Food Allergy. Curr. Allergy Asthma Rep..

[94] Astolfi A., Cipriani F., Messelodi D., De Luca M., Indio V., Di Chiara C., Giannetti A., Ricci L., Neri I., Patrizi A. (2021). Filaggrin Loss-of-Function Mutations Are Risk Factors for Severe Food Allergy in Children with Atopic Dermatitis. J. Clin. Med..

[95] Johansson E.K., Bergström A., Kull I., Lind T., Söderhäll C., van Hage M., Wickman M., Ballardini N., Wahlgren C.F. (2017). IgE sensitization in relation to preschool eczema and filaggrin mutation. J. Allergy Clin. Immunol..

[96] Brown S.J., Asai Y., Cordell H.J., Campbell L.E., Zhao Y., Liao H., Northstone K., Henderson J., Alizadehfar R., Ben-Shoshan M. (2011). Loss-of-function variants in the filaggrin gene are a significant risk factor for peanut allergy. J. Allergy Clin. Immunol..

[97] Van Ginkel C.D., Flokstra-de Blok B.M.J., Kollen B.J., Kukler J., Koppelman G.H., Dubois A.E.J. (2015). Loss-of-function variants of the filaggrin gene are associated with clinical reactivity to foods. Allergy.

[98] Kalb B., Marenholz I., Jeanrenaud A.C.S.N., Meixner L., Arnau-Soler A., Rosillo-Salazar O.D., Ghauri A., Cibin P., Blümchen K., Schlags R. (2022). Filaggrin loss-of-function mutations are associated with persistence of egg and milk allergy. J. Allergy Clin. Immunol..

[99] Liang Y., Chang C., Lu Q. (2016). The Genetics and Epigenetics of Atopic Dermatitis-Filaggrin and Other Polymorphisms. Clin. Rev. Allergy Immunol..

[100] Buelow L.M., Hoji A., Tat K., Schroeder-Carter L.M., Carroll D.J., Cook-Mills J.M. (2021). Mechanisms for Alternaria alternata Function in the Skin During Induction of Peanut Allergy in Neonatal Mice With Skin Barrier Mutations. Front. Allergy.

[101] Saunders S.P., Goh C.S., Brown S.J., Palmer C.N., Porter R.M., Cole C., Campbell L.E., Gierlinski M., Barton G.J., Schneider G. (2013). Tmem79/Matt is the matted mouse gene and is a predisposing gene for atopic dermatitis in human subjects. J. Allergy Clin. Immunol..

[102] Bergmann S., von Buenau B., Vidal-y-Sy S., Hafek M., Wladykovski E., Houdek P., Lezius S., Duplan H., Bäsler K., Dähnhardt-Pfeifer S. (2020). Claudin-1 decrease impacts epidermal barrier function in atopic dermatitis lesions dose-dependently. Sci. Rep..

[103] Brewer M.G., Anderson E.A., Pandya R.P., De Benedetto A., Yoshida T., Hilimire T.A., Martinez-Sobrido L., Beck L.A., Miller B.L. (2020). Peptides Derived from the Tight Junction Protein CLDN1 Disrupt the Skin Barrier and Promote Responsiveness to an Epicutaneous Vaccine. J. Investig. Dermatol..

[104] Zaniboni M.C., Samorano L.P., Orfali R.L., Aoki V. (2016). Skin barrier in atopic dermatitis: Beyond filaggrin. Bras Dermatol..

[105] Brown S.J. (2021). What Have We Learned from GWAS for Atopic Dermatitis?. J. Investig. Dermatol..

[106] Tamari M., Hirota T. (2014). Genome-wide association studies of atopic dermatitis. J. Dermatol..

[107] Nakahara T., Kido-Nakahara M., Tsuji G., Furue M. (2021). Basics and recent advances in the pathophysiology of atopic dermatitis. J. Dermatol..

[108] Yang G., Seok J.K., Kang H.C., Cho Y.Y., Lee H.S., Lee J.Y. (2020). Skin Barrier Abnormalities and Immune Dysfunction in Atopic Dermatitis. Int. J. Mol. Sci..

[109] Thyssen J.P., Kezic S. (2014). Causes of epidermal filaggrin reduction and their role in the pathogenesis of atopic dermatitis. J. Allergy Clin. Immunol..

[110] Celebi Sozener Z., Ozdel Ozturk B., Cerci P., Turk M., Gorgulu Akin B., Akdis M., Altiner S., Ozbey U., Ogulur I., Mitamura Y. (2022). Epithelial barrier hypothesis: Effect of the external exposome on the microbiome and epithelial barriers in allergic disease. Allergy.

[111] To T., Zhu J., Stieb D., Gray N., Fong I., Pinault L., Jerrett M., Robichaud A., Ménard R., van Donkelaar A. (2020). Early life exposure to air pollution and incidence of childhood asthma, allergic rhinitis and eczema. Eur. Respir. J..

[112] Patella V., Florio G., Palmieri M., Bousquet J., Tonacci A., Giuliano A., Gangemi S. (2020). Atopic dermatitis severity during exposure to air pollutants and weather changes with an Artificial Neural Network (ANN) analysis. Pediatr. Allergy Immunol..

[113] Oh I., Lee J., Ahn K., Kim J., Kim Y.M., Sun Sim C., Kim Y. (2018). Association between particulate matter concentration and symptoms of atopic dermatitis in children living in an industrial urban area of South Korea. Environ. Res..

[114] Kim Y.M., Kim J., Jung K., Eo S., Ahn K. (2018). The effects of particulate matter on atopic dermatitis symptoms are influenced by weather type: Application of spatial synoptic classification (SSC). Int. J. Hyg. Environ. Health.

[115] Dijkhoff I.M., Drasler B., Karakocak B.B., Petri-Fink A., Valacchi G., Eeman M., Rothen-Rutishauser B. (2020). Impact of airborne particulate matter on skin: A systematic review from epidemiology to in vitro studies. Part. Fibre Toxicol..

[116] Ngoc L.T.N., Park D., Lee Y., Lee Y.-C. (2017). Systematic Review and Meta-Analysis of Human Skin Diseases Due to Particulate Matter. Int. J. Environ. Res. Public Health.

[117] Piao M.J., Ahn M.J., Kang K.A., Ryu Y.S., Hyun Y.J., Shilnikova K., Zhen A.X., Jeong J.W., Choi Y.H., Kang H.K. (2018). Particulate matter 2.5 damages skin cells by inducing oxidative stress, subcellular organelle dysfunction, and apoptosis. Arch. Toxicol..

[118] Kim B.E., Kim J., Goleva E., Berdyshev E., Lee J., Vang K.A., Lee U.H., Han S., Leung S., Hall C.F. (2021). Particulate matter causes skin barrier dysfunction. JCI Insight.

[119] Yee M.S., Hii L.W., Looi C.K., Lim W.M., Wong S.F., Kok Y.Y., Tan B.K., Wong C.Y., Leong C.O. (2021). Impact of Microplastics and Nanoplastics on Human Health. Nanomaterials.

[120] Wright C., Iyer A.K., Wang L., Wu N., Yakisich J.S., Rojanasakul Y., Azad N. (2017). Effects of titanium dioxide nanoparticles on human keratinocytes. Drug Chem. Toxicol..

[121] Cork M.J., Danby S.G., Vasilopoulos Y., Hadgraft J., Lane M.E., Moustafa M., Guy R.H., Macgowan A.L., Tazi-Ahnini R., Ward S.J. (2009). Epidermal barrier dysfunction in atopic dermatitis. J. Investig. Dermatol..

[122] Tanzer J., Meng D., Ohsaki A., Caldwell J.M., Mingler M.K., Rothenberg M.E., Oyoshi M.K. (2022). Laundry detergent promotes allergic skin inflammation and esophageal eosinophilia in mice. PLoS ONE.

[123] Leoty-Okombi S., Gillaizeau F., Leuillet S., Douillard B., Le Fresne-Languille S., Carton T., De Martino A., Moussou P., Bonnaud-Rosaye C., André V. (2021). Effect of Sodium Lauryl Sulfate (SLS) Applied as a Patch on Human Skin Physiology and Its Microbiota. Cosmetics.

[124] Xian M., Wawrzyniak P., Rückert B., Duan S., Meng Y., Sokolowska M., Globinska A., Zhang L., Akdis M., Akdis C.A. (2016). Anionic surfactants and commercial detergents decrease tight junction barrier integrity in human keratinocytes. J. Allergy Clin. Immunol..

[125] Khosrowpour Z., Ahmad Nasrollahi S., Ayatollahi A., Samadi A., Firooz A. (2019). Effects of four soaps on skin trans-epidermal water loss and erythema index. J. Cosmet. Dermatol..

[126] Guertler A., Moellhoff N., Schenck T.L., Hagen C.S., Kendziora B., Giunta R.E., French L.E., Reinholz M. (2020). Onset of occupational hand eczema among healthcare workers during the SARS-CoV-2 pandemic: Comparing a single surgical site with a COVID-19 intensive care unit. Contact Dermat..

[127] Kendziora B., Guertler A., Ständer L., Frey S., French L.E., Wollenberg A., Reinholz M. (2020). Evaluation of hand hygiene and onset of hand eczema after the outbreak of SARS-CoV-2 in Munich. Eur. J. Dermatol..

[128] Jabbar-Lopez Z.K., Ung C.Y., Alexander H., Gurung N., Chalmers J., Danby S., Cork M.J., Peacock J.L., Flohr C. (2021). The effect of water hardness on atopic eczema, skin barrier function: A systematic review, meta-analysis. Clin. Exp. Allergy.

[129] Perkin M.R., Craven J., Logan K., Strachan D., Marrs T., Radulovic S., Campbell L.E., MacCallum S.F., McLean W.H., Lack G. (2016). Association between domestic water hardness, chlorine, and atopic dermatitis risk in early life: A population-based cross-sectional study. J. Allergy Clin. Immunol..

[130] Engebretsen K.A., Bager P., Wohlfahrt J., Skov L., Zachariae C., Nybo Andersen A.M., Melbye M., Thyssen J.P. (2017). Prevalence of atopic dermatitis in infants by domestic water hardness and season of birth: Cohort study. J. Allergy Clin. Immunol..

[131] Danby S.G., Brown K., Wigley A.M., Chittock J., Pyae P.K., Flohr C., Cork M.J. (2018). The Effect of Water Hardness on Surfactant Deposition after Washing and Subsequent Skin Irritation in Atopic Dermatitis Patients and Healthy Control Subjects. J. Investig. Dermatol..

[132] Prescott S.L., Larcombe D.L., Logan A.C., West C., Burks W., Caraballo L., Levin M., Etten E.V., Horwitz P., Kozyrskyj A. (2017). The skin microbiome: Impact of modern environments on skin ecology, barrier integrity, and systemic immune programming. World Allergy Organ. J..

[133] Koh L.F., Ong R.Y., Common J.E. (2022). Skin microbiome of atopic dermatitis. Allergol. Int..

[134] Altunbulakli C., Reiger M., Neumann A.U., Garzorz-Stark N., Fleming M., Huelpuesch C., Castro-Giner F., Eyerich K., Akdis C.A., Traidl-Hoffmann C. (2018). Relations between epidermal barrier dysregulation and Staphylococcus species-dominated microbiome dysbiosis in patients with atopic dermatitis. J. Allergy Clin. Immunol..

[135] Nakatsuji T., Gallo R.L. (2019). The role of the skin microbiome in atopic dermatitis. Ann. Allergy Asthma Immunol..

[136] Paller A.S., Kong H.H., Seed P., Naik S., Scharschmidt T.C., Gallo R.L., Luger T., Irvine A.D. (2019). The microbiome in patients with atopic dermatitis. J. Allergy Clin. Immunol..

[137] Tauber M., Balica S., Hsu C.Y., Jean-Decoster C., Lauze C., Redoules D., Viodé C., Schmitt A.M., Serre G., Simon M. (2016). Staphylococcus aureus density on lesional and nonlesional skin is strongly associated with disease severity in atopic dermatitis. J. Allergy Clin. Immunol..

[138] Kim J., Kim B.E., Ahn K., Leung D.Y.M. (2019). Interactions Between Atopic Dermatitis and Staphylococcus aureus Infection: Clinical Implications. Allergy Asthma Immunol. Res..

[139] Simpson E.L., Villarreal M., Jepson B., Rafaels N., David G., Hanifin J., Taylor P., Boguniewicz M., Yoshida T., De Benedetto A. (2018). Patients with Atopic Dermatitis Colonized with Staphylococcus aureus Have a Distinct Phenotype and Endotype. J. Investig. Dermatol..

[140] Czarnowicki T., Krueger J.G., Guttman-Yassky E. (2014). Skin barrier and immune dysregulation in atopic dermatitis: An evolving story with important clinical implications. J. Allergy Clin. Immunol. Pract..

[141] Clausen M.L., Edslev S.M., Andersen P.S., Clemmensen K., Krogfelt K.A., Agner T. (2017). Staphylococcus aureus colonization in atopic eczema and its association with filaggrin gene mutations. Br. J. Dermatol..

[142] Geoghegan J.A., Irvine A.D., Foster T.J. (2018). Staphylococcus aureus and Atopic Dermatitis: A Complex and Evolving Relationship. Trends Microbiol..

[143] Forbes-Blom E., Camberis M., Prout M., Tang S.C., Le Gros G. (2012). Staphylococcal-derived superantigen enhances peanut induced Th2 responses in the skin. Clin. Exp. Allergy.

[144] Jones A.L., Curran-Everett D., Leung D.Y.M. (2016). Food allergy is associated with Staphylococcus aureus colonization in children with atopic dermatitis. J. Allergy Clin. Immunol..

[145] Tsilochristou O., du Toit G., Sayre P.H., Roberts G., Lawson K., Sever M.L., Bahnson H.T., Radulovic S., Basting M., Plaut M. (2019). Association of Staphylococcus aureus colonization with food allergy occurs independently of eczema severity. J. Allergy Clin. Immunol..

[146] Galand C., Leyva-Castillo J.M., Yoon J., Han A., Lee M.S., McKenzie A.N.J., Stassen M., Oyoshi M.K., Finkelman F.D., Geha R.S. (2016). IL-33 promotes food anaphylaxis in epicutaneously sensitized mice by targeting mast cells. J. Allergy Clin. Immunol..

[147] Bartnikas L.M., Gurish M.F., Burton O.T., Leisten S., Janssen E., Oettgen H.C., Beaupré J., Lewis C.N., Austen K.F., Schulte S. (2013). Epicutaneous sensitization results in IgE-dependent intestinal mast cell expansion and food-induced anaphylaxis. J. Allergy Clin. Immunol..

[148] Leyva-Castillo J.M., Hener P., Jiang H., Li M. (2013). TSLP produced by keratinocytes promotes allergen sensitization through skin and thereby triggers atopic march in mice. J. Investig. Dermatol..

[149] Khodoun M.V., Tomar S., Tocker J.E., Wang Y.H., Finkelman F.D. (2018). Prevention of food allergy development and suppression of established food allergy by neutralization of thymic stromal lymphopoietin, IL-25, and IL-33. J. Allergy Clin. Immunol..

[150] Tamagawa-Mineoka R., Okuzawa Y., Masuda K., Katoh N. (2014). Increased serum levels of interleukin 33 in patients with atopic dermatitis. J. Am. Acad Dermatol..

[151] Imai Y. (2019). Interleukin-33 in atopic dermatitis. J. Dermatol. Sci..

[152] Savinko T., Matikainen S., Saarialho-Kere U., Lehto M., Wang G., Lehtimäki S., Karisola P., Reunala T., Wolff H., Lauerma A. (2012). IL-33 and ST2 in atopic dermatitis: Expression profiles and modulation by triggering factors. J. Investig. Dermatol..

[153] Chinthrajah S., Cao S., Liu C., Lyu S.C., Sindher S.B., Long A., Sampath V., Petroni D., Londei M., Nadeau K.C. (2019). Phase 2a randomized, placebo-controlled study of anti-IL-33 in peanut allergy. JCI Insight.

[154] Muto T., Fukuoka A., Kabashima K., Ziegler S.F., Nakanishi K., Matsushita K., Yoshimoto T. (2014). The role of basophils and proallergic cytokines, TSLP and IL-33, in cutaneously sensitized food allergy. Int. Immunol..

[155] Borowczyk J., Shutova M., Brembilla N.C., Boehncke W.H. (2021). IL-25 (IL-17E) in epithelial immunology and pathophysiology. J. Allergy Clin. Immunol..

[156] Cherrier M., Cerf-Bensussan N. (2019). Scratching Beneath the Surface: Linking Skin Pathology with Food Allergy. Immunity.

[157] von Moltke J., Ji M., Liang H.E., Locksley R.M. (2016). Tuft-cell-derived IL-25 regulates an intestinal ILC2-epithelial response circuit. Nature.

[158] Lee J.B., Chen C.Y., Liu B., Mugge L., Angkasekwinai P., Facchinetti V., Dong C., Liu Y.J., Rothenberg M.E., Hogan S.P. (2016). IL-25 and CD4(+) TH2 cells enhance type 2 innate lymphoid cell-derived IL-13 production, which promotes IgE-mediated experimental food allergy. J. Allergy Clin. Immunol..

[159] De Benedetto A., Kubo A., Beck L.A. (2012). Skin barrier disruption: A requirement for allergen sensitization?. J. Investig. Dermatol..

[160] Oyoshi M.K., Larson R.P., Ziegler S.F., Geha R.S. (2010). Mechanical injury polarizes skin dendritic cells to elicit a T(H)2 response by inducing cutaneous thymic stromal lymphopoietin expression. J. Allergy Clin. Immunol..

[161] Kim J., Kim B.E., Lee J., Han Y., Jun H.Y., Kim H., Choi J., Leung D.Y.M., Ahn K. (2016). Epidermal thymic stromal lymphopoietin predicts the development of atopic dermatitis during infancy. J. Allergy Clin. Immunol..

[162] Soumelis V., Reche P.A., Kanzler H., Yuan W., Edward G., Homey B., Gilliet M., Ho S., Antonenko S., Lauerma A. (2002). Human epithelial cells trigger dendritic cell mediated allergic inflammation by producing TSLP. Nat. Immunol..

[163] Zhu T.H., Zhu T.R., Tran K.A., Sivamani R.K., Shi V.Y. (2018). Epithelial barrier dysfunctions in atopic dermatitis: A skin-gut-lung model linking microbiome alteration and immune dysregulation. Br. J. Dermatol..

[164] Segaud J., Yao W., Marschall P., Daubeuf F., Lehalle C., German B., Meyer P., Hener P., Hugel C., Flatter E. (2022). Context-dependent function of TSLP and IL-1β in skin allergic sensitization and atopic march. Nat. Commun..

[165] Hussain M., Borcard L., Walsh K.P., Pena Rodriguez M., Mueller C., Kim B.S., Kubo M., Artis D., Noti M. (2018). Basophil-derived IL-4 promotes epicutaneous antigen sensitization concomitant with the development of food allergy. J. Allergy Clin. Immunol..

[166] Noti M., Kim B.S., Siracusa M.C., Rak G.D., Kubo M., Moghaddam A.E., Sattentau Q.A., Comeau M.R., Spergel J.M., Artis D. (2014). Exposure to food allergens through inflamed skin promotes intestinal food allergy through the thymic stromal lymphopoietin-basophil axis. J. Allergy Clin. Immunol..

[167] Smeekens J.M., Kulis M.D. (2021). Mouse Models of Food Allergy in the Pursuit of Novel Treatment Modalities. Front. Allergy.

[168] Tham E.H., Leung D.Y. (2019). Mechanisms by Which Atopic Dermatitis Predisposes to Food Allergy and the Atopic March. Allergy Asthma Immunol. Res..

[169] Tordesillas L., Goswami R., Benedé S., Grishina G., Dunkin D., Järvinen K.M., Maleki S.J., Sampson H.A., Berin M.C. (2014). Skin exposure promotes a Th2-dependent sensitization to peanut allergens. J. Clin. Investig..

[170] Yagami A., Aihara M., Ikezawa Z., Hide M., Kishikawa R., Morita E., Chinuki Y., Fukutomi Y., Urisu A., Fukushima A. (2017). Outbreak of immediate-type hydrolyzed wheat protein allergy due to a facial soap in Japan. J. Allergy Clin. Immunol..

[171] Weidinger S., Beck L.A., Bieber T., Kabashima K., Irvine A.D. (2018). Atopic dermatitis. Nat. Rev. Dis. Prim..

[172] He H., Del Duca E., Diaz A., Kim H.J., Gay-Mimbrera J., Zhang N., Wu J., Beaziz J., Estrada Y., Krueger J.G. (2021). Mild atopic dermatitis lacks systemic inflammation and shows reduced nonlesional skin abnormalities. J. Allergy Clin. Immunol..

[173] Wollenberg A., Kinberger M., Arents B., Aszodi N., Avila Valle G., Barbarot S., Bieber T., Brough H.A., Calzavara Pinton P., Christen-Zäch S. (2022). European guideline (EuroGuiDerm) on atopic eczema-part II: Non-systemic treatments and treatment recommendations for special AE patient populations. J. Eur. Acad Dermatol. Venereol..

[174] Kang S.-Y., Um J.-Y., Chung B.-Y., Lee S.-Y., Park J.-S., Kim J.-C., Park C.-W., Kim H.-O. (2022). Moisturizer in Patients with Inflammatory Skin Diseases. Medicina.

[175] Schachner L.A., Hebert A.A., Anneke Andriessen A., Benjamin L.T., Ana M., Duarte A.M., Goldberg N., Kwong P.C., Rico T.S., Eichenfield L.F. (2019). A Global Review on the Risk Factors and Management of Early Atopic Dermatitis in Children Ages 0 to 2 Years Old. J. Drugs Dermatol..

[176] Elias P.M. (2022). Optimizing emollient therapy for skin barrier repair in atopic dermatitis. Ann. Allergy Asthma Immunol..

[177] Fluhr J.W., Darlenski R., Lachmann N., Baudouin C., Msika P., De Belilovsky C., Hachem J.P. (2012). Infant epidermal skin physiology: Adaptation after birth. Br. J. Dermatol..

[178] Simpson E.L., Chalmers J.R., Hanifin J.M., Thomas K.S., Cork M.J., McLean W.H., Brown S.J., Chen Z., Chen Y., Williams H.C. (2014). Emollient enhancement of the skin barrier from birth offers effective atopic dermatitis prevention. J. Allergy Clin. Immunol..

[179] Horimukai K., Morita K., Narita M., Kondo M., Kitazawa H., Nozaki M., Shigematsu Y., Yoshida K., Niizeki H., Motomura K. (2014). Application of moisturizer to neonates prevents development of atopic dermatitis. J. Allergy Clin. Immunol..

[180] Glatz M., Jo J.H., Kennedy E.A., Polley E.C., Segre J.A., Simpson E.L., Kong H.H. (2018). Emollient use alters skin barrier and microbes in infants at risk for developing atopic dermatitis. PLoS ONE.

[181] Yonezawa K., Haruna M., Matsuzaki M., Shiraishi M., Kojima R. (2018). Effects of moisturizing skincare on skin barrier function and the prevention of skin problems in 3-month-old infants: A randomized controlled trial. J. Dermatol..

[182] McClanahan D., Wong A., Kezic S., Samrao A., Hajar T., Hill E., Simpson E.L. (2019). A randomized controlled trial of an emollient with ceramide and filaggrin-associated amino acids for the primary prevention of atopic dermatitis in high-risk infants. J. Eur. Acad Dermatol. Venereol.

[183] Dissanayake E., Tani Y., Nagai K., Sahara M., Mitsuishi C., Togawa Y., Suzuki Y., Nakano T., Yamaide F., Ohno H. (2019). Skin Care and Synbiotics for Prevention of Atopic Dermatitis or Food Allergy in Newborn Infants: A 2 × 2 Factorial, Randomized, Non-Treatment Controlled Trial. Int. Arch. Allergy Immunol..

[184] Skjerven H.O., Rehbinder E.M., Vettukattil R., LeBlanc M., Granum B., Haugen G., Hedlin G., Landrø L., Marsland B.J., Rudi K. (2020). Skin emollient and early complementary feeding to prevent infant atopic dermatitis (PreventADALL): A factorial, multicentre, cluster-randomised trial. Lancet.

[185] Chalmers J.R., Haines R.H., Bradshaw L.E., Montgomery A.A., Thomas K.S., Brown S.J., Ridd M.J., Lawton S., Simpson E.L., Cork M.J. (2020). Daily emollient during infancy for prevention of eczema: The BEEP randomised controlled trial. Lancet.

[186] Bradshaw L.E., Wyatt L.A., Brown S.J., Haines R.H., Montgomery A.A., Perkin M.R., Lawton S., Sach T.H., Chalmers J.R., Ridd M.J. (2022). Emollients for prevention of atopic dermatitis: 5-year findings from the BEEP randomized trial. [published online ahead of print, 2022 Oct 19]. Allergy.

[187] Skjerven H.O., Lie A., Vettukattil R., Rehbinder E.M., LeBlanc M., Asarnoj A., Carlsen K.H., Despriee A.W., Färdig M., Wärnberg Gerdin S. (2022). Early food intervention and skin emollients to prevent food allergy in young children (PreventADALL): A factorial, multicentre, cluster-randomised trial. Lancet.

[188] Lowe A.J., Su J.C., Allen K.J., Abramson M.J., Cranswick N., Robertson C.F., Forster D., Varigos G., Hamilton S., Kennedy R. (2018). A randomized trial of a barrier lipid replacement strategy for the prevention of atopic dermatitis and allergic sensitization: The PEBBLES pilot study. Br. J. Dermatol..

[189] Lowe A., Su J., Tang M., Lodge C.J., Matheson M., Allen K.J., Varigos G., Sasi A., Cranswick N., Hamilton S. (2019). PEBBLES study protocol: A randomised controlled trial to prevent atopic dermatitis, food allergy and sensitisationin infants with a family history of allergic disease using a skin barrier improvement strategy. BMJ. Open.

[190] Eichner B., Michaels L.A.C., Branca K., Ramsey K., Mitchell J., Morris C.D., Fagnan L.J., Dolor R.J., Elder N., Hahn D.L. (2020). A Community-based Assessment of Skin Care, Allergies, and Eczema (CASCADE): An atopic dermatitis primary prevention study using emollients-protocol for a randomized controlled trial. Trials.

[191] Sindher S., Alkotob S.S., Shojinaga M.N., Brough H.A., Bahnson H.T., Chan S., Lack G., Leung D.Y.M., Nadeau K.C. (2020). Pilot study measuring transepidermal water loss (TEWL) in children suggests trilipid cream is more effective than a paraffin-based emollient. Allergy.

[192] Sindher S., Alkotob S.S., Shojinaga M.N., Hamilton R., Chan S., Cao S., Bahnson H.T., Brough H.A., Lack G., Leung D.Y.M. (2020). Increases in plasma IgG4/IgE with trilipid vs paraffin/petrolatum-based emollients for dry skin/eczema. Pediatr. Allergy Immunol..

[193] Kelleher M.M., Cro S., Van Vogt E., Cornelius V., Lodrup Carlsen K.C., Ove Skjerven H., Rehbinder E.M., Lowe A., Dissanayake E., Shimojo N. (2021). Skincare interventions in infants for preventing eczema and food allergy: A cochrane systematic review and individual participant data meta-analysis. Clin. Exp. Allergy.

[194] Marrs T., Perkin M.R., Logan K., Craven J., Radulovic S., McLean W.H.I., Versteeg S.A., van Ree R., Lack G., Flohr C. (2020). Bathing frequency is associated with skin barrier dysfunction and atopic dermatitis at three months of age. J. Allergy Clin. Immunol. Pract..

[195] Perkin M.R., Logan K., Marrs T., Radulovic S., Craven J., Boyle R.J., Chalmers J.R., Williams H.C., Versteeg S.A., van Ree R. (2021). Association of frequent moisturizer use in early infancy with the development of food allergy. J. Allergy Clin. Immunol..

[196] Danby S.G., Chalmers J., Brown K., Williams H.C., Cork M.J. (2016). A functional mechanistic study of the effect of emollients on the structure and function of the skin barrier. Br. J. Dermatol..

[197] Cooke A., Cork M.J., Victor S., Campbell M., Danby S., Chittock J., Lavender T. (2016). Olive Oil, Sunflower Oil or no Oil for Baby Dry Skin or Massage: A Pilot, Assessor-blinded, Randomized Controlled Trial (the Oil in Baby SkincaRE [OBSeRvE] Study). Acta Dermatol. Venereol..

[198] Katibi O.S., Cork M.J., Flohr C., Danby S.G. (2022). Moisturizer therapy in prevention of atopic dermatitis and food allergy: To use or disuse?. Ann. Allergy Asthma Immunol..

[199] Zhong Y., Samuel M., van Bever H., Tham E.H. (2022). Emollients in infancy to prevent atopic dermatitis: A systematic review and meta-analysis. Allergy.

[200] Priyadarshi M., Balachander B., Gupta S., Sankar M.J. (2022). Topical emollient application in term healthy newborns: A systematic review. J. Glob. Health.

[201] Furue M. (2020). Regulation of Filaggrin, Loricrin, and Involucrin by IL-4, IL-13, IL-17A, IL-22, AHR, and NRF2: Pathogenic Impli-cations in Atopic Dermatitis. Int. J. Mol. Sci.

[202] Fukuie T., Nomura I., Horimukai K., Manki A., Masuko I., Futamura M., Narita M., Ohzeki T., Matsumoto K., Saito H. (2010). Proactive treatment appears to decrease serum immunoglobulin-E levels in patients with severe atopic dermatitis. Br. J. Dermatol..

[203] Miyaji Y., Yang L., Yamamoto-Hanada K., Narita M., Saito H., Ohya Y. (2020). Earlier aggressive treatment to shorten the duration of eczema in infants resulted in fewer food allergies at 2 years of age. J. Allergy Clin. Immunol. Pract..

[204] Yamamoto-Hanada K., Kobayashi T., Williams H.C., Mikami M., Saito-Abe M., Morita K., Natsume O., Sato M., Iwama M., Miyaji Y. (2018). Early aggressive intervention for infantile atopic dermatitis to prevent development of food allergy: A multicenter, investigator-blinded, randomized, parallel group controlled trial (PACI Study)-protocol for a randomized controlled trial. Clin. Transl. Allergy.

[205] Rowley G.G., MacNeill S.J., Ridd M.J. (2022). Emollient satisfaction questionnaire: Validation study in children with eczema. Clin. Exp. Dermatol..

[206] Al-Zuhairy S.A.S., Kadhum W.R., Alhijjaj M., Kadhim M.M., Al-Janabi A.S., Salman A.W., Al-Sharifi H.K.R., Khadom A.A. (2022). Development and Evaluation of Biocompatible Topical Petrolatum-liquid Crystal Formulations with Enhanced Skin Permeation Properties. J. Oleo Sci..

[207] Danby S.G., Andrew P.V., Kay L.J., Pinnock A., Chittock J., Brown K., Williams S.F., Cork M.J. (2022). Enhancement of stratum corneum lipid structure improves skin barrier function and protects against irritation in adults with dry, eczema-prone, skin. Br. J. Dermatol..

[208] van Zuuren E.J., Fedorowicz Z., Christensen R., Lavrijsen A., Arents B.W.M. (2017). Emollients and moisturisers for eczema. Cochrane Database Syst. Rev..

[209] Danby S.G., Draelos Z.D., Gold L.F.S., Cha A., Vlahos B., Aikman L., Sanders P., Wu-Linhares D., Cork M.J. (2022). Vehicles for atopic dermatitis therapies: More than just a placebo. J. Dermatol. Treat..

[210] Celleno L. (2018). Topical urea in skincare: A review. Dermatol. Ther..

[211] Karagounis T.K., Gittler J.K., Rotemberg V., Morel K.D. (2019). Use of "natural" oils for moisturization: Review of olive, coconut, and sunflower seed oil. Pediatr. Dermatol..

[212] Vanessa V.V., Wan Ahmad Kammal W.S.L., Lai Z.W., How K.N. (2022). A Review of Moisturizing Additives for Atopic Dermatitis. Cosmetics.

[213] Ryczaj K., Dumycz K., Spiewak R., Feleszko W. (2022). Contact allergens in moisturizers in preventative emollient therapy–A systematic review. Clin. Transl. Allergy.

[214] Łoś-Rycharska E., Gołębiewski M., Grzybowski T., Rogalla-Ładniak U., Krogulska A. (2020). The microbiome and its impact on food allergy and atopic dermatitis in children. Postep. Dermatol. Alergol..

[215] Sinha S., Lin G., Ferenczi K. (2021). The skin microbiome and the gut-skin axis. Clin. Dermatol..

[216] Bunyavanich S. (2019). Food allergy: Could the gut microbiota hold the key?. Nat. Rev. Gastroenterol. Hepatol..

[217] Gołębiewski M., Łoś-Rycharska E., Sikora M., Grzybowski T., Gorzkiewicz M., Krogulska A. (2021). Mother’s milk microbiome shaping fecal and skin microbiota in infants with food allergy and atopic dermatitis: A pilot analysis. Nutrients.

[218] van den Elsen L.W.J., Garssen J., Burcelin R., Verhasselt V. (2019). Shaping the Gut Microbiota by Breastfeeding: The Gateway to Allergy Prevention?. Front. Pediatr..

[219] Dzidic M., Mira A., Artacho A., Abrahamsson T.R., Jenmalm M.C., Collado M.C. (2020). Allergy development is associated with consumption of breastmilk with a reduced microbial richness in the first month of life. Pediatr. Allergy Immunol..

[220] Ho N.T., Li F., Lee-Sarwar K.A., Tun H.M., Brown B.P., Pannaraj P.S., Bender J.M., Azad M.B., Thompson A.L., Weiss S.T. (2018). Meta-analysis of effects of exclusive breastfeeding on infant gut microbiota across populations. Nat. Commun..

[221] Kim H., Sitarik A.R., Woodcroft K., Johnson C.C., Zoratti E. (2019). Birth Mode, Breastfeeding, Pet Exposure, and Antibiotic Use: Associations With the Gut Microbiome and Sensitization in Children. Curr. Allergy Asthma Rep..

[222] Sitarik A.R., Bobbitt K.R., Havstad S.L., Fujimura K.E., Levin A.M., Zoratti E.M., Kim H., Woodcroft K.J., Wegienka G., Ownby D.R. (2017). Breast Milk Transforming Growth Factor β Is Associated With Neonatal Gut Microbial Composition. J. Pediatr. Gastroenterol. Nutr..

[223] Johnson C.C., Ownby D.R. (2017). The infant gut bacterial microbiota and risk of pediatric asthma and allergic diseases. Transl. Res..

[224] Rajani P.S., Seppo A.E., Järvinen K.M. (2018). Immunologically Active Components in Human Milk and Development of Atopic Disease, With Emphasis on Food Allergy, in the Pediatric Population. Front. Pediatr..

[225] Home–ClinicalTrials.gov. https://clinicaltrials.gov/.

[226] Nagel-Wolfrum K., Möller F., Penner I., Baasov T., Wolfrum U. (2016). Targeting Nonsense Mutations in Diseases with Translational Read-Through-Inducing Drugs (TRIDs). BioDrugs.

[227] Campofelice A., Lentini L., Di Leonardo A., Melfi R., Tutone M., Pace A., Pibiri I. (2019). Strategies against Nonsense: Oxadiazoles as Translational Readthrough-Inducing Drugs (TRIDs). Int. J. Mol. Sci..

[228] Stout T.E., McFarland T., Mitchell J.C., Appukuttan B., Timothy Stout J. (2014). Recombinant filaggrin is internalized and processed to correct filaggrin deficiency. J. Investig. Dermatol..

[229] Kim Y., Lim K.M. (2021). Skin barrier dysfunction and filaggrin. Arch. Pharm. Res..

[230] Peltonen J.M., Pylkkänen L., Jansén C.T., Volanen I., Lehtinen T., Laihia J.K., Leino L. (2014). Three randomised phase I/IIa trials of 5% cis-urocanic acid emulsion cream in healthy adult subjects and in patients with atopic dermatitis. Acta Dermatol. Venereol..

[231] Papp K., Szepietowski J.C., Kircik L., Toth D., Kuligowski M.E., Venturanza M.E., Sun K., Simpson E.L. (2020). Efficacy and Safety of Ruxolitinib Cream for the Treatment of Atopic Dermatitis: Results from Two Phase 3, Randomized, Double-Blind Studies. SKIN J. Cutan. Med..

[232] Nakagawa H., Nemoto O., Igarashi A., Saeki H., Kaino H., Nagata T. (2020). Delgocitinib ointment, a topical Janus kinase inhibitor, in adult patients with moderate to severe atopic dermatitis: A phase 3, randomized, double-blind, vehicle-controlled study and an open-label, long-term extension study. J. Am. Acad. Dermatol..

[233] Kim B.S., Sun K., Papp K., Venturanza M., Nasir A., Kuligowski M.E. (2020). Effects of ruxolitinib cream on pruritus and quality of life in atopic dermatitis: Results from a phase 2, randomized, dose-ranging, vehicle- and active-controlled study. J. Am. Acad Dermatol..

[234] McLornan D.P., Pope J.E., Gotlib J., Harrison C.N. (2021). Current and future status of JAK inhibitors. Lancet.

[235] Sideris N., Paschou E., Bakirtzi K., Kiritsi D., Papadimitriou I., Tsentemeidou A., Sotiriou E., Vakirlis E. (2022). New and Upcoming Topical Treatments for Atopic Dermatitis: A Review of the Literature. J. Clin. Med..

[236] Peppers J., Paller A.S., Maeda-Chubachi T., Wu S., Robbins K., Gallagher K., Kraus J.E. (2019). A phase 2, randomized dose-finding study of tapinarof (GSK2894512 cream) for the treatment of atopic dermatitis. J. Am. Acad Dermatol..

[237] Paller A.S., Stein Gold L., Soung J., Tallman A.M., Rubenstein D.S., Gooderham M. (2021). Efficacy and patient-reported outcomes from a phase 2b, randomized clinical trial of tapinarof cream for the treatment of adolescents and adults with atopic dermatitis. J. Am. Acad. Dermatol..

[238] Furue M., Uchi H., Mitoma C., Hashimoto-Hachiya A., Tanaka Y., Ito T., Tsuji G. (2019). Implications of tryptophan photoproduct FICZ in oxidative stress and terminal differentiation of keratinocytes. G Ital. Dermatol. Venereol..

[239] Kiyomatsu-Oda M., Uchi H., Morino-Koga S., Furue M. (2018). Protective role of 6-formylindolo[3,2-b]carbazole (FICZ), an endogenous ligand for arylhydrocarbon receptor, in chronic mite-induced dermatitis. J. Dermatol. Sci..

[240] Lee J., Song K.M., Jung C.H. (2021). Diosmin restores the skin barrier by targeting the aryl hydrocarbon receptor in atopic dermatitis. Phytomedicine.

[241] Jia T., Qiao W., Yao Q., Wu W., Kaku K. (2019). Treatment with Docosahexaenoic Acid Improves Epidermal Keratinocyte Differentiation and Ameliorates Inflammation in Human Keratinocytes and Reconstructed Human Epidermis Models. Molecules.

[242] Czarnowicki T., Dohlman A.B., Malik K., Antonini D., Bissonnette R., Chan T.C., Zhou L., Wen H.C., Estrada Y., Xu H. (2018). Effect of short-term liver X receptor activation on epidermal barrier features in mild to moderate atopic dermatitis: A randomized controlled trial. Ann. Allergy Asthma Immunol..

[243] Bielach-Bazyluk A., Zbroch E., Mysliwiec H., Rydzewska-Rosolowska A., Kakareko K., Flisiak I., Hryszko T. (2021). Sirtuin 1 and Skin: Implications in Intrinsic and Extrinsic Aging–A Systematic Review. Cells.

[244] Jin T., Park K.Y., Seo S.J. (2017). Adiponectin Upregulates Filaggrin Expression via SIRT1-Mediated Signaling in Human Normal Keratinocytes. Ann. Dermatol..

[245] Che D.N., Cho B.O., Shin J.Y., Kang H.J., Kim J., Choi J., Jang S.I. (2020). Anti-atopic dermatitis effects of hydrolyzed celery extract in mice. J. Food Biochem..

[246] Che D.N., Cho B.O., Shin J.Y., Kang H.J., Kim J.S., Oh H., Kim Y.S., Jang S.I. (2019). Apigenin Inhibits IL-31 Cytokine in Human Mast Cell and Mouse Skin Tissues. Molecules.

[247] Egawa G., Kabashima K. (2016). Multifactorial skin barrier deficiency and atopic dermatitis: Essential topics to prevent the atopic march. J. Allergy Clin. Immunol..

[248] Furue M. (2020). Regulation of Skin Barrier Function via Competition between AHR Axis versus IL-13/IL-4-JAK-STAT6/STAT3 Axis: Pathogenic and Therapeutic Implications in Atopic Dermatitis. J. Clin. Med..

[249] Renert-Yuval Y., Guttman-Yassky E. (2020). New treatments for atopic dermatitis targeting beyond IL-4/IL-13 cytokines. Ann. Allergy Asthma Immunol..

[250] Moyle M., Cevikbas F., Harden J.L., Guttman-Yassky E. (2019). Understanding the immune landscape in atopic dermatitis: The era of biologics and emerging therapeutic approaches. Exp. Dermatol..

[251] Szalus K., Trzeciak M., Nowicki R.J. (2020). JAK-STAT Inhibitors in Atopic Dermatitis from Pathogenesis to Clinical Trials Results. Microorganisms.

[252] He H., Guttman-Yassky E. (2019). JAK Inhibitors for Atopic Dermatitis: An Update. Am. J. Clin. Dermatol..

[253] Tanimoto Y., Shinozaki Y., Yamamoto Y., Katsuda E., Taniai-Riya K., Toyoda K., Kakimoto K., Kimoto Y., Amano W., Konishi N. (2018). A novel JAK inhibitor JTE-052 reduces skin inflammation and ameliorates chronic dermatitis in rodent models: Comparison with conventional therapeutic agents. Exp. Dermatol..

[254] Singh R., Heron C.E., Ghamrawi R.I., Strowd L.C., Feldman S.R. (2020). Emerging Role of Janus Kinase Inhibitors for the Treatment of Atopic Dermatitis. Immunotargets.

[255] Nguyen H.L., Anderson K.R., Tollefson M.M. (2019). New and emerging therapies for pediatric atopic dermatitis. Paediatr. Drugs.

[256] Clarysse K., Pfaff C., Marquardt Y., Huth L., Kortekaas K., Kluwig D., Lüscher B., Gutermuth J., Baron J. (2019). JAK1/3 inhibition preserves epidermal morphology in full-thickness 3D skin models of atopic dermatitis and psoriasis. J. Eur. Acad Dermatol. Venereol.

[257] Furue M., Nakahara T. (2020). Revival of AHR Agonist for the Treatment of Atopic Dermatitis: Tapinarof. Curr. Treat. Options Allergy.

[258] Bissonnette R., Stein Gold L., Rubenstein D.S., Tallman A.M., Armstrong A. (2021). Tapinarof in the treatment of psoriasis: A review of the unique mechanism of action of a novel therapeutic aryl hydrocarbon receptor-modulating agent. J. Am. Acad Dermatol..

